# Exposure to Excess Phenobarbital Negatively Influences the Osteogenesis of Chick Embryos

**DOI:** 10.3389/fphar.2016.00349

**Published:** 2016-09-30

**Authors:** Yu Yan, Xin Cheng, Ren-Hao Yang, He Li, Jian-Long Chen, Zheng-Lai Ma, Guang Wang, Manli Chuai, Xuesong Yang

**Affiliations:** ^1^Division of Histology and Embryology, Key Laboratory for Regenerative Medicine of the Ministry of Education, Medical College, Jinan UniversityGuangzhou, China; ^2^Division of Cell and Developmental Biology, University of DundeeDundee, UK

**Keywords:** phenobarbital, chick embryos, chondrogenesis, mineralization, angiogenesis, osteogenesis

## Abstract

Phenobarbital is an antiepileptic drug that is widely used to treat epilepsy in a clinical setting. However, a long term of phenobarbital administration in pregnant women may produce side effects on embryonic skeletogenesis. In this study, we aim to investigate the mechanism by which phenobarbital treatment induces developmental defects in long bones. We first determined that phenobarbital treatment decreased chondrogenesis and inhibited the proliferation of chondrocytes in chick embryos. Phenobarbital treatment also suppressed mineralization in both *in vivo* and *in vitro* long bone models. Next, we established that phenobarbital treatment delayed blood vessel invasion in a cartilage template, and this finding was supported by the down-regulation of vascular endothelial growth factor in the hypertrophic zone following phenobarbital treatment. Phenobarbital treatment inhibited tube formation and the migration of human umbilical vein endothelial cells. In addition, it impaired angiogenesis in chick yolk sac membrane model and chorioallantoic membrane model. In summary, phenobarbital exposure led to shortened lengths of long bones during embryogenesis, which might result from inhibiting mesenchyme differentiation, chondrocyte proliferation, and delaying mineralization by impairing vascular invasion.

## Introduction

Phenobarbital (PB), an antiepileptic drug (AEDs), is a sedative hypnotic barbiturate and an anticonvulsant drug. It is commonly used to control their seizures in pregnant women with epilepsy (Lowe, 2001). It is also used to treat bipolar disorder, migraine prophylaxis, cancer and neuropathic pain (Wlodarczyk et al., 2012). Importantly, AEDs are used to avoid complications in pregnant women with epilepsy. Because pregnant women still develop status epilepticus, the mortality rates of the mother and the baby will increase if she stops taking AEDs (Ahir and Pratten, 2014). In addition, epilepsy in pregnancy could lead to fetal intracranial hemorrhage and heart rate alterations (Johnson et al., 1989). However, the side effects include classic osteomalacia (Hahn et al., 1978), craniofacial growth retardation, cleft palate and congenital heart defects (Azarbayjani and Danielsson, 1998; Holmes et al., 2004) when the concentration of AEDs is excessive. Therefore, it is absolutely necessary to monitor AEDs application in clinical settings to reduce fetal mortality and to avoid teratogenicity (Ornoy, 2006). Although as a kind of commonly used AEDs clinically, there has been little evidence gathered on the side effects of PB on embryogenesis, except that a few studies on the mechanisms of PB-induced embryonic cardiovascular malformation (Schmitz et al., 2010b; Ahir and Pratten, 2014), it remains unclear about the explicit mechanism how PB treatment affects bone development during embryogenesis.

Vertebrate skeletal development occurs by two distinct mechanisms: intramembranous and endochondral ossification (Stickens et al., 2004). Intramembranous ossification occurs in the formation of flat bones such as the skull vault, cranium and clavicle (Ornitz and Marie, 2002). Most mammalian bones form through endochondral ossification such as long bone of the limbs, basal bones of the skull, vertebrae, ribs and the pelvis (Cheng et al., 2016).

As the prophase of endochondral ossification, chondrogenesis is regulated by a large number of signaling molecules, such as Sox9 (Zhou et al., 2015). Chondrocytes in the hyaline cartilage begin to form a specialized extracellular matrix that synthesizes type II collagen (Araldi and Schipani, 2010), and the chondrocytes near the ends of the cartilaginous template proliferate rapidly, while those in the center of the template exit the cell cycle, undergo hypertrophy and produce type X collagen to replace type II collagen (Dao et al., 2012). The programmed death of hypertrophic chondrocytes and blood vessel invasion indicate that ossification has begun. The cartilage is gradually replaced by bone while the bone marrow forms (Cheng et al., 2014). Along with bone development, bone marrow extends toward the epiphyseal growth plate, which is made up of well-demarcated zones of cells (Hall and Miyake, 1992). The resting or reserve zone (RZ) near the ends of the cartilaginous template supplies cells to the proliferating zone (PZ), in which the cells are arranged in columns and are always proliferating; then, those cells begin to differentiate into hypertrophic chondrocytes to form the hypertrophic zone (HZ).

Being a rigid and tightly compacted organ, bone is also highly vascularized (Simon and Keith, 2008). Angiogenesis plays a crucial role in bone formation and repair (Provot and Schipani, 2007). A well-established vascular system in bone tissue is indispensable for endochondral ossification (Kanczler and Oreffo, 2008). A key feature of the endochondral ossification process is that the cartilage template will be gradually replaced by bone tissues along with blood vessel invasion (Kronenberg, 2003; Provot and Schipani, 2005). Next the increasing numbers of blood vessels introduce more osteoblast progenitors which increase endochondral ossification. Regarded as a coupling process, osteogenesis-angiogenesis is essential for keeping homeostasis during bone development, and it may also aid in finding the target of therapies for bone regeneration and repair. A large number of signaling molecules are involved in angiogenesis to regulate the production of new blood vessels from a pre-existing vasculature (Polverini, 2002; Carmeliet, 2003). A vital angiogenesis regulator in the cartilaginous template replacement is vascular endothelial growth factor (VEGFA), which is released by the late hypertrophic chondrocytes and induces blood vessels to invade the cartilage model (Pfander et al., 2004; Zelzer and Olsen, 2005). The receptors of VEGFA, VEGF-R1 (Flt-1), and VEGF-R2 (KDR/Flk-1) are also important for angiogenesis during embryonic osteogenesis (Shibuya, 2006). Many other signaling molecules, such as hypoxia-inducible factor 1α (HIF-1α) and parathyroid hormone-related protein (PTHrP), play important roles during mutually dependent osteogenesis and angiogenesis (Bentovim et al., 2012; Kigami et al., 2013).

In this study, we employed chick embryos as model to explore the effects of PB on bone development during embryogenesis *in vivo*, and then combined *in vitro* cell cultures to investigate the role of angiogenesis in PB-interfered osteogenesis, to adequately assess the true impact of PB on skeletogenesis.

## Materials and methods

### Embryo manipulation

Fertilized Leghorn eggs were obtained from the Avian Farm of the South China Agriculture University (Guangzhou, China) and were incubated in a humidified incubator (Hamburger and Hamilton) (Misske et al., 2007). PB (98% purity, Merck) were dissolved in 0.9% sterile saline and then stored in 4°C. The chick embryos were exposed to different concentrations of PB (0.04, 0.4, or 4 mM) or 0.9% sterile saline (control) for 15.5 days. Briefly, approximately 200 μL of 0.9% sterile saline or 0.04, 0.4, or 4 mM PB was carefully injected into a small hole made in the air chamber of the egg every other day from day 1.5 until day 17. The surviving embryos were harvested for skeleton staining (*n* = 6 for each group).

### Alcian blue and alizarin red staining

To visualize the skeleton, the chick embryos were stained with alcian blue and alizarin red as previously described (Schmitz et al., 2010a). Day-17 chick embryos were freed from adherent tissue, fixed in 95% ethanol for 3 days, stained for cartilage with alcian blue and counterstained for bone with alizarin red (Solarbio, Beijing, China). Long-bone tissues were carefully photographed using a stereomicroscope (Olympus MVX10, Japan). The length of the alizarin red-stained portion of each radius, ulna, tibia and phalanx was quantified using Image Pro-Plus 5.0 (Media Cybernetics).

### Phalange explant cultures

The fertilized eggs were incubated for 14 days; then, the growth plates of phalanges were isolated and randomly used for control (0.9% sterile saline) or PB treatment (0.4 or 1.6 mM). The growth plates were cultured in F-12 (Myclone, USA) supplemented with 10% fetal bovine serum (FBS, Gibco, Gaithersburg, MD, USA) containing PB or 0.9% sterile saline (control) at 37°C and 5% CO_2_ (Galaxy S, RS Biotech, UK). After incubation for 72 h, the cultured growth plates were examined using semi-quantitative RT-PCR analysis (*n* = 3 for each group).

### Angiogenesis assessment of yolk sac membrane (YSM)

Fertilized eggs were incubated for 2.5 days and then placed into a sterilized glass dish with the YSM facing upward (*n* = 6 for each group). Two silicone rings were placed on top of the leading edge of the blood vessels marked with ink to indicate the starting position of the YSM within the ring. 50 μL of 0.9% sterile saline (control) was introduced into the ring located on the left side of the YSM, marked in black. Fifty microliter of 0.4 or 1.6 mM PB was introduced into the ring marked in red on the right side of the same embryo. The extent of the expansion of the blood vessel plexus inside the silicone rings was determined and photographed after incubation for 12–36 h. The density of blood vessels in the YSM was analyzed using Image Pro-Plus 5.0 software. The blood vessel density is expressed as the percentage of the blood vessel area in the whole stereomicroscopic field (He et al., 2014). The extended distance of blood vessels was also quantified. Some YSMs were also embedded in paraffin wax, serially sectioned at 5 μm (Leica RM2126RT, Germany) and stained with hematoxylin & eosin (H&E). The rest of the YSMs were harvested for RNA isolation.

### Angiogenesis assessment in chorioallantoic membrane (CAM)

Chick embryos were incubated until day 7.5 (*n* = 3 for each group), when the CAM is well developed. The embryos were treated with PB (0.4 or 1.6 mM) or 0.9% sterile saline (control) for 48 h, and all surviving embryos were harvested for analysis. The CAM and accompanying blood vessels in the control and PB-treated embryos were photographed using a Canon Powershot SX130 IS digital camera (12.1 M Pixels). The blood vessel density was quantified as described above for assessing angiogenesis in the YSM. The CAMs were also harvested for different biochemical assays as described below.

### Histological analysis and immunofluorescence staining

Seventeen-day-old embryos treated with PB were harvested and fixed in 4% paraformaldehyde (PFA). The phalanges of the embryos were decalcified using a 10% EDTA solution in 1 mM PBS (pH 7.4) for 7 days at 4°C and were then embedded in paraffin. The samples were serially sectioned at 5 μm thicknesses using a microtome (Leica RM2126RT, Germany). Longitudinal sections of these bones were produced and further stained with H&E using a standard protocol for histological observations (Schmitz et al., 2010b) (*n* = 4 for each group). The extent of apoptosis in the bone tissues was detected by TUNEL analysis using an *in situ* Cell Death Detection Kit (Roche, Switzerland) (*n* = 4 for each group). The staining was performed according to the manufacturer's protocol and was adapted for bone section labeling. Immunofluorescence staining was performed on some sections of the phalanges using a monoclonal primary antibody against p-Histone H3 (pH3, 1:400, Santa Cruz Biotechnology) or a rabbit polyclonal PCNA (1:100, Santa Cruz Biotechnology) at 4°C overnight, followed by Alexa Fluor 555-labeled anti-rabbit IgG secondary antibody (1:1000, Invitrogen, CA, USA) (*n* = 5.6 for each group). The sections were counterstained with 4′-6-diamidino-2-phenylindole (DAPI, 5 μg/mL; Life Tech, USA) to reveal the nuclei and were finally photographed using an Olympus IX51 microscope. Histomorphometry was performed on TUNEL-, pH3- and PCNA-immunofluorescent sections of phalange growth plates using Image Pro-Plus 5.0 software. Cell proliferation was evaluated as a percentage of pH3^+^ cells or PCNA^+^ cells relative to the corresponding cells of the control groups. Cell apoptosis was evaluated as a percentage of TUNEL^+^ cells relative to the corresponding cells of the control groups.

### Cell culture, immunofluorescence staining, and F-actin staining

Human umbilical vein endothelial cells (HUVECs, a kind gift from Zhi Huang's lab) and MC3T3-E1 cells (a mouse pre-osteoblastic cell line that was a gift from Chao Wan's lab) were cultured in a humidified incubator at 5% CO_2_ and 37°C in 6-well plates (1 × 10^6^ cells/ml) containing DMEM/F12 (Myclone, USA) supplemented with 10% FBS; cells were exposed to PB (0.4 or 1.6 mM) or control (0.9% sterile saline). The cells were photographed using an inverted fluorescence microscope (Nikon, Ti-u, Japan) linked to NIS-Elements F3.2 software. After incubation for 24 h, these cultures were incubated with p-Histone H3 primary antibody (pH3, 1:400, Santa Cruz Biotechnology) at 4°C overnight (*n* = 6 for each group). Then, Alexa Fluor 555-labeled anti-rabbit IgG secondary antibody was used for visualizing the primary antibody. For F-actin detection, cultured cells were stained using phalloidin-Alexa Fluor 555 (1:500, Invitrogen) at room temperature for 2 h. All the cells were counterstained with DAPI at room temperature for 1 h. Cell proliferation was evaluated as a percentage of pH3^+^ HUVECs or pH3^+^ MC3T3-E1 cells relative to the corresponding cells of the control groups.

### Cell counting kit-8 (CCK8) assay

The viability of HUVECs and MC3T3-E1 cells was assessed using a modified CCK8 assay (Dojindo Molecular Technologies, Japan). All of the cells were cultured in 96-well plates (2.5 × 10^4^ cells/ml) as described above and were exposed to PB (0.1, 0.2, 0.4, 0.8, or 1.6 mM) or the control (0.9% sterile saline). After 24 h, 10 μL of CCK8 (5 g/L) was added into the 96-well plates, followed by incubation for 4 h at 37°C. The absorbance values were measured at 450 nm using a Bio-Rad Model 450 Microplate Reader (Bio-Rad, CA, USA). Cell viability was indirectly established using the ratio of the absorbance value of PB-treated cells relative to the control (*n* = 6 for each group).

### Morphometry of mesenchymal differentiation of cultured cells

The mesenchymal cells were dissected from 4.5-day (Hamburger and Hamilton Stage 23, HH23) chick embryos and were insolated as previously described (Ahrens et al., 1977; San Antonio and Tuan, 1986; Delise and Tuan, 2002). Briefly, limb buds were dissected from HH23 chick embryos and treated with trypsin (0.25%; Life Tech, USA), the ectoderm was removed and limb buds were gently dissociated into single cells. The cells were cultured in 6-well plates (2.5 × 10^4^ cells/ml) containing DMEM supplemented with 10% FBS and were exposed to either PB (0.4 or 1.6 mM) or the control (0.9% sterile saline). Following treatment with different concentrations of PB for 2 weeks, the cultures were fixed in 95% ethanol for 20 min and then stained with 1% toluidine blue at room temperature overnight to demonstrate the chondrogenic differentiation.

### Micromass cell culture and morphometry of chondrogenic matrix production by cultured cells

Micromass cultures were produced from limb bud mesenchymal cells as previously described (Delise and Tuan, 2002). Briefly, limb buds were dissected from HH23 chick embryos and were treated with trypsin; the ectoderm was removed and limb buds were gently dissociated into single cells. The cells were suspended in DMEM (Life Tech, USA) with 10% FBS at a density of 2.0 × 10^7^ cells/mL and were spotted as 10 μL droplets per well on a 6-well plate. After 3 h of pre-incubation, all the wells were flooded with 500 μL of culture medium. The cells were incubated at 37°C and 5% CO_2_ in an incubator (Galaxy S, RS Biotech, UK). PB (0.4 or 1.6 mM) dissolved in DMEM with 10% FBS was introduced on the second day after plating. The control cultures received 0.9% sterile saline only. The culture medium was changed every 2 days.After incubation for 3 days, the cultured cells were fixed in 95% ethanol and then stained with 1% alcian blue dye (pH 1.0) overnight at room temperature. The alcian blue-stained cartilage nodules that formed in the absence or presence of PB were photographed using an inverted microscope (Nikon Eclipse Ti-U, Japan). The average size (area) of the chondrogenic nodules was digitized as total stain intensity/nodule number (*n* = 4 for each group).

### Mineralization of MC3T3-E1 cultures

MC3T3-E1 cells were cultured as described above for the micromass cell cultures. After treatment with PB (0.4 or 1.6 mM) for 7 days, the cultures were fixed in 95% ethanol for 20 min and then were stained with 2% alizarin red dye (pH 4.2) at room temperature overnight to detect the calcium deposits (*n* = 3 for each group).

### Tube formation assay

Each well of a 12-well plate was coated with 200 μL of a mixture of Matrigel (BD Biosciences, USA); then, the plate was incubated at 37°C for 30 min to promote gelling. HUVECs were resuspended in DMEM/F12 medium with 10% FBS in the absence or presence of PB (0.4 or 1.6 mM), and the final volume each well was 1 ml. Photographs were taken after incubation for 4–8 h using an inverted microscope (Nikon Eclipse Ti-U, Japan) at the middle of each well. The average number of tubules was calculated using the examinations of six separate microscopic fields. Tube formation in the presence of PB was compared to tube formation in media with 0.9% sterile saline as the control or the control vector (*n* = 3 for each group).

### Scratch-wound assay

HUVECs were seeded in 6-well plates with DMEM/F12 medium. At confluence, a wound was induced by scratching the monolayer with a 1-mL pipette tip. The cells were then washed 3 times with sterile PBS. HUVECs were incubated in serum-free DMEM/F12 medium with PB (0.4 or 1.6 mM) or 0.9% sterile saline (control) at 5% CO_2_. Images were acquired at 0, 12, 24, and 36 h post-scratching. The images were taken using an inverted microscope (Nikon Eclipse Ti-U, Japan) (*n* = 6 for each group).

### Semi-quantitative RT-PCR

Total RNA was extracted from the cells and tissues using a Trizol kit (Invitrogen, USA). First-strand cDNA was synthesized to a final volume of 25 μL using a SuperScript RIII first-strand kit (Invitrogen, USA). Following reverse transcription, PCR amplification of the cDNA was performed using chick-specific primers. The primers sequences are provided in Supplementary Figure 1. The PCR reactions were performed using a Bio-Rad S1000TM Thermal cycler (Bio-Rad, USA) as previously described (Ahir and Pratten, 2014). The resolved PCR products were visualized using a transilluminator (SYNGENE, UK), and photographs were captured using a computer-assisted gel documentation system (SYNGENE). The intensity of the fluorescently stained bands was measured and normalized using Image Pro-Plus.

### Data analysis

Data analyses and construction of statistical charts were performed using Graphpad Prism 5 (Graphpad Software, CA, USA). The results were presented as mean ± SD. All comparisons among groups were made using ANOVA or Student's *t*-test.

## Ethics statements

This study was approved by the Institutional Animal Care and Use Committee in Jinan University Medical College, Guangzhou, China and all efforts were made to minimize suffering.

## Results

### PB treatment delays endochondral ossification and shortens long bones

**Figure 1:**
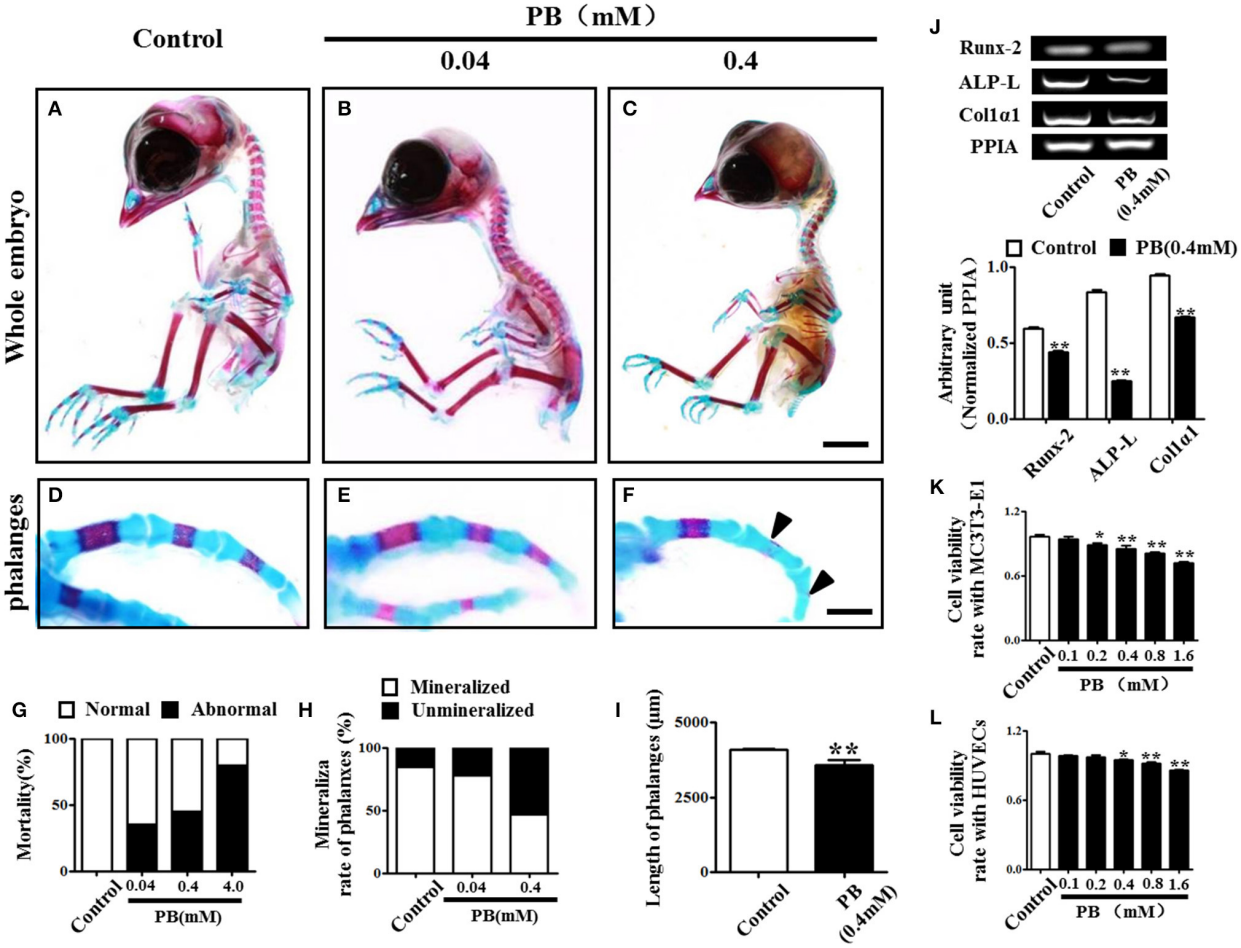
**Alcian blue and alizarin red staining of the phalanx and assessment of cell viability following PB treatment**. Early chick embryos (day 1.5) were treated with PB (0.04, 0.4, or 4 mM) for 15.5 days; then, the skeletal structures were stained with alcian blue and alizarin red dyes. **(A–C)** Control embryo **(A)**, embryos treated with 0.04 mM **(B)** or 0.4 mM PB **(C)**. **(D–F)** Representative images of phalanges from control **(D)** and PB-treated **(E,F)** chick embryos at day 17. **(G)** Bar graph showing embryonic mortality when embryos were exposed to different concentrations of PB. **(H)** Bar graph comparing the rate of alizarin red^+^ phalanges in all of the phalanges between the control and PB-treated groups. **(I)** Bar graph comparing the length of phalanges between the control and 0.4 mM PB-treated groups. **(J)** Semi-quantitative RT-PCR and bar graphs showing the expression of Runx-2, ALP-L and Col1α1 in phalanges following 0.4 mM PB treatment. **(K,L)** Bar graphs showing the viability of MC3T3-E1 cells **(K)** and HUVECs **(L)** after 0.9% sterile saline (control) or PB (0.1–1.6 mM) treatment for 24 h. Scale bars = 1 cm in **(A,B)** and 2 mm in **(D–F)**. *P < 0.05, **P < 0.01.

To investigate the effect of PB on skeletal development, we performed alcian blue/alizarin red staining to examine skeletal development in detail (Figures 1A–C, Supplementary Figures 2A,B) and observed that exposing chick embryos to 0.4 mM PB caused a marked defect in the ossification of several cartilage-based structures. In the axial skeleton, the defects in endochondral ossification were evident in the vertebral column (Supplementary Figures 2A',B'). Simultaneously, in the appendicular skeleton at the level of the limbs, 0.4 mM PB treatment impaired endochondral ossification centers in the phalanges (Figures 1D,F,H), the radius and ulna (Supplementary Figures 2A”,B”), and the tibia (Supplementary Figures 2A”',B”'). The ossification of phalanges, however, was indistinguishable between the control embryos and the embryos treated with 0.04 mM PB, suggesting that lower concentrations of PB do not affect ossification (Figures 1E,H). We did not show the embryos treated with 4 mM PB because of their high mortality rates (Figure 1G). The length of the phalanges was measured in control and PB (0.4 mM) treated group (Figure 1I, Supplementary Table 1). For the ulna, the rate of alizarin red^+^ staining was measured between control and PB treatments and statistical analyzed (Supplementary Figure 2C, Supplementary Table 1). For the radius, the rate of alizarin red^+^ staining was measured between control and PB treatments and statistical analyzed (Supplementary Figure 2D, Supplementary Table 1). For the tibia, the rate of alizarin red^+^ staining was measured between control and PB treatments and statistical analyzed (Supplementary Figure 2E, Supplementary Table 1). The length of ulna was measured in control and PB (0.4 mM) treated group (Supplementary Figure 2F, Supplementary Table 1). The length of radius was measured in control and PB (0.4 mM) treated group (Supplementary Figure 2G, Supplementary Table 1). The length of tibia was measured in control and PB (0.4 mM) treated group (Supplementary Figure 2H, Supplementary Table 1). RT-PCR data showed that PB treatment down-regulated osteogenesis-related genes, including Runx-2, ALP-L and Col1α1 (Figure 1J, Supplementary Table 1).

Next, we used MC3T3-E1 cells and HUVECs to test the effect of PB on cell viability. MC3T3-E1 cell viability was inhibited by PB in a dose-dependent manner in comparison to that of the control (Figure 1K, Supplementary Table 2), as was HUVEC viability in comparison to that of the control group (Figure 1L, Supplementary Table 2). These results imply that PB treatment during embryogenesis shortened embryonic long bones and inhibited mineralization *in vivo*.

### PB treatment inhibits chondrogenesis

**Figure 2:**
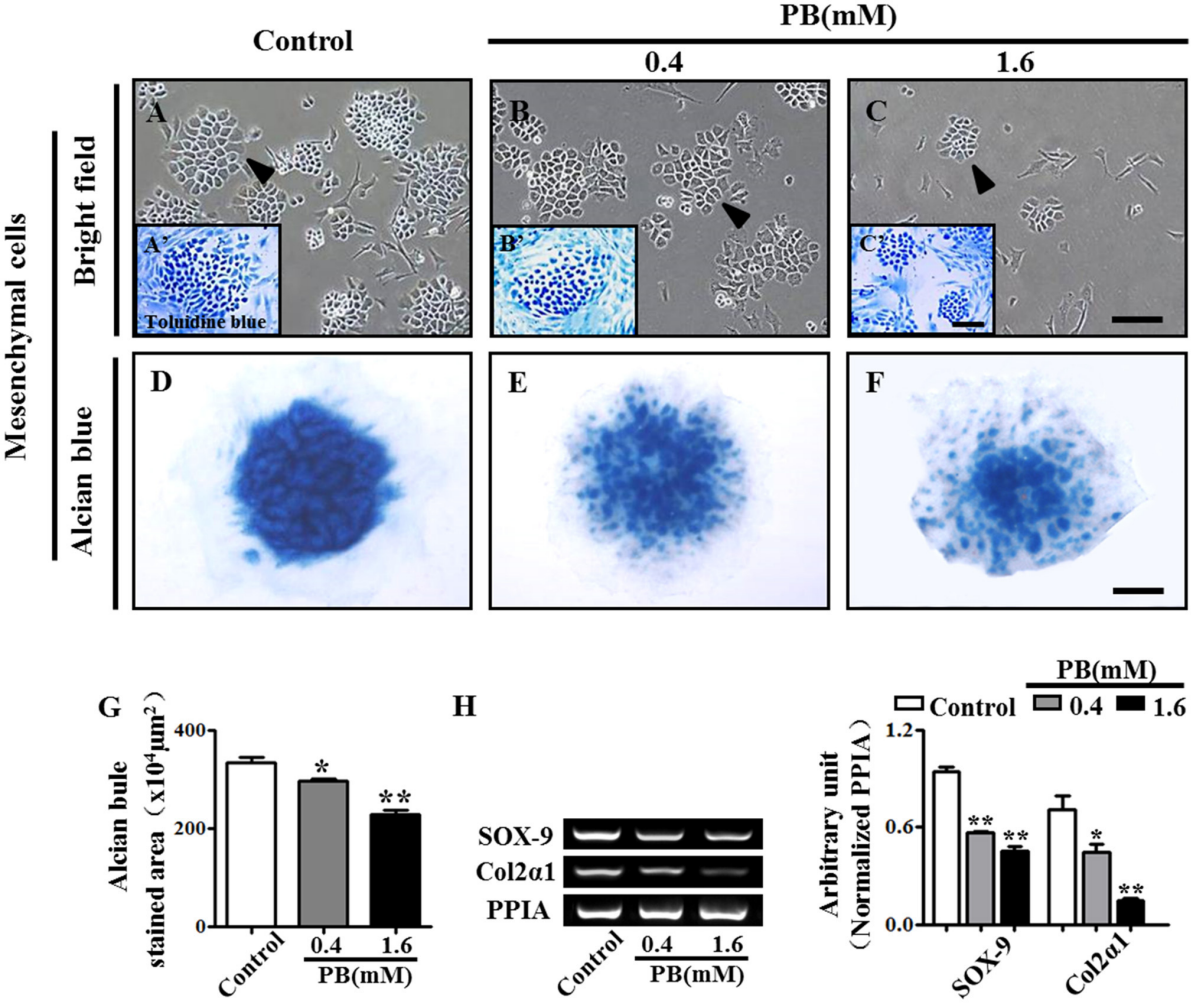
**PB treatment effects on the chondrogenesis of chick mesenchyme *in vitro***. Alcian blue staining was performed on the micromass cultures treated with various concentrations of PB. The limb buds of HH23 chick embryos were dissected into mesenchymal cells and were cultured in presence of different concentrations of PB. **(A–C)** Representative light micrographs of the mesenchyme cell cultures treated with 0.9% sterile saline (control, **A**), 0.4 mM PB **(B)**, or 1.6 mM PB **(C)** for 2 weeks. **(A'–C')** Representative images of toluidine blue-stained mesenchyme cells, which were incubated with 0.9% sterile saline (control, **A')**, 0.4 mM PB **(B')**, or 1.6 mM PB **(C')** for 2 weeks. **(D–F)** Representative micrographs of alcian blue-stained micromass cultures, which were incubated with 0.9% sterile saline (control, **D**), 0.4 mM PB **(E)**, or 1.6 mM PB **(F)** for 72 h. **(G)** Bar chart showing the average size (area) of chondrogenic nodules formed in the presence of 0.9% sterile saline or PB after 72-h incubation. **(H)** Semi-quantitative RT-PCR and bar graph showing the expression of Sox-9 and Col2α1 in the cell mass culture following PB treatment. Scale bars = 300 μm in **(A–C)**, 150 μm in **(A'–C')**, and 500 μm in **(D–F)**. *P < 0.05, **P < 0.01.

To investigate whether PB could affect mesenchyme differentiation, the limb buds of HH23 chick embryos were dissected into single cells and were then cultured in a monolayer *in vitro* for 2 weeks. The cells were stained with toluidine blue to verify that they were chondrocytes that had differentiated from mesenchyme (arrowheads in Figures 2A–C). In the presence of PB, the number of chondrocytes was reduced in comparison with that of the control group, suggesting that PB inhibits chondrogenesis. To further confirm this result, the high-density micromass culture system of limb bud mesenchymal cells was used. In this model, chondrogenesis is initiated when the mesenchymal cells start to condense and aggregate to form large nodules. These nodules appear morphologically similar to cartilage. After 3 days of culture, the nodules began to produce an extracellular matrix (Mello and Tuan, 1999). The size of alcian blue-positive cartilaginous nodules in PB was smaller than those of the control group (Figures 2D–F). We further measured the alcian blue-positive area in the absence or presence of PB and found that it was consistent with the results from the alcian blue staining (Figure 2G, Supplementary Table 3). RT-PCR showed that PB exposure down-regulated chondrogenesis-related genes, including SOX-9 (*n* = 3 for each group) and Col2α1 (Figure 2H, Supplementary Table 3). These observations indicate that PB treatment triggered a delay in chondrogenesis.

### PB treatment expands the hypertrophic zone and suppresses chondrocyte proliferation

**Figure 3:**
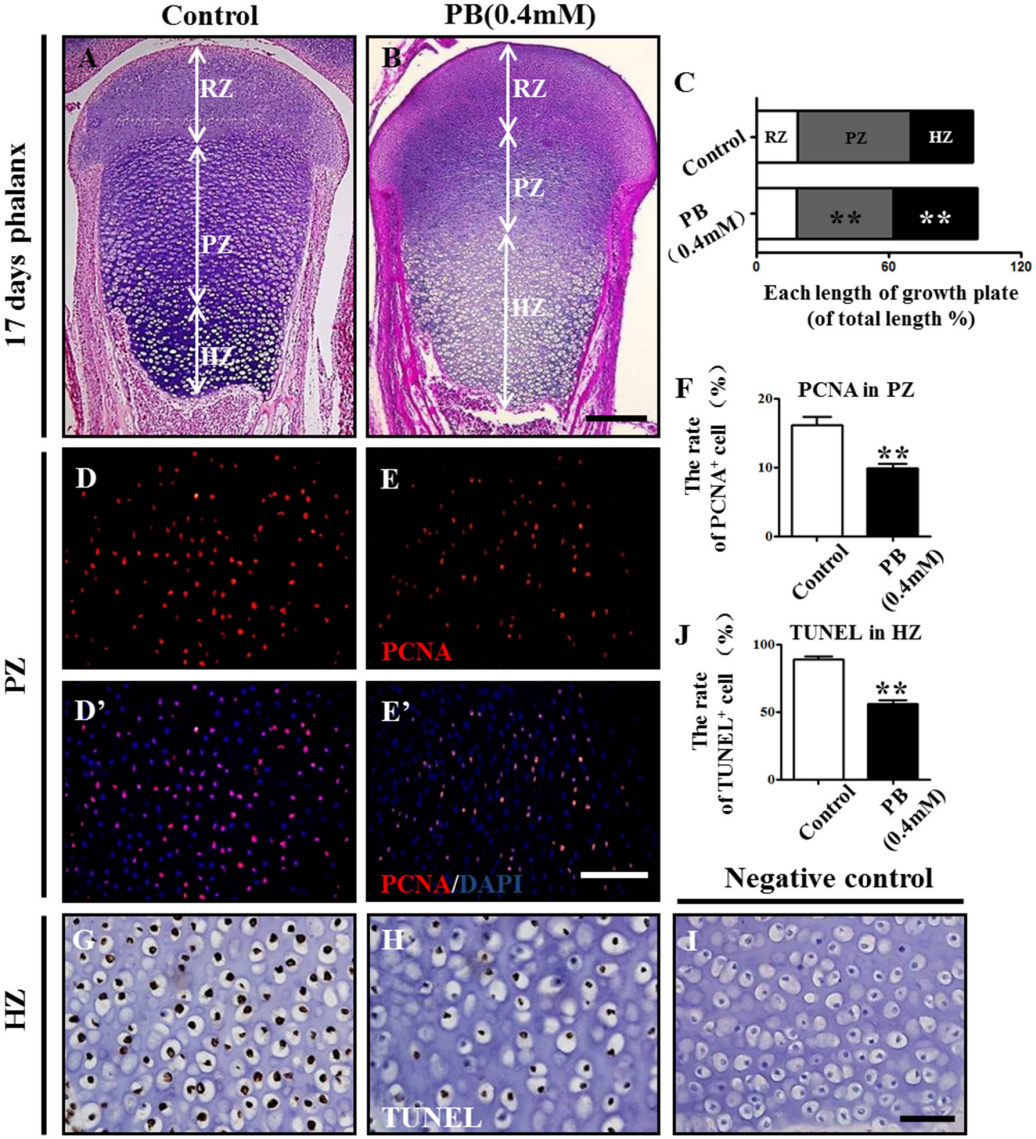
**PB treatment effects on the length and cell cycles of growth plates in phalanges**. Histological analysis of epiphyseal growth plates in phalanges of day 17 chick embryos exposed to PB. **(A,B)** Representative images of H&E stained phalanges sections from control **(A)** and PB treatment **(B)** groups at day 17. **(C)** Bar graph comparing the length of each zone in the growth plate in the control and PB groups. **(D–E**'**):** PCNA immunofluorescence of phalanges in control **(D,D**'**)** and PB-treated embryos **(E,E**'**)**. **(F)** Bar graph comparing the proportion of PCNA^+^ cells in PZ in control and PB-treated vertical sections of phalanges. **(G,H)** HZ of control **(G)** and PB-treated phalanges **(H)** stained with TUNEL to indicate apoptotic cells. **(I)** Representative images of the negative control of TUNEL in the HZ of the growth plate. **(J)** Bar graph comparing apoptotic chondrocytes of HZ in control and PB-treated phalanges. Scale bars = 600 μm in **(A,B)**, 100 μm in **(D–E**'**)** and 25 μm in **(G–I)**. ** *P* < 0.05.

To explore the mechanism of delayed endochondral ossification in PB-treated chick embryos, we first performed H&E staining in the growth plate. The H&E stained phalanx sections showed that the HZ in the PB groups was longer than in the control group. Moreover, the PZ in the PB groups was shorter than in the control group (Figures 3A,B). For the growth plate, the rates in different zones are shown in Figure 3C and Supplementary Table 4.

To exclude the possibility that the expansion of HZ resulted from increased chondrocyte proliferation, we examined the proliferation rate of chondrocytes in the PZ or RZ using pH3 or PCNA immunofluorescence staining. The results revealed fewer pH3^+^ or PCNA^+^ cells in the RZ and PZ of the growth plates exposed to PB (Figures 3D–F,D'–E', Supplementary Figures 3A–F,A'–D', Supplementary Table 4). These results suggest that expanded HZ was not associated with increased cell proliferation, but it may be related to the decreased long-bone length in the PB-treated 17-day-old chick embryos.

To determine whether the expanded HZ was associated with the reduction of cell death, we examined apoptosis in RZ and PZ using the TUNEL assay. There was no apparent difference in the RZ or PZ between the PB and control groups (Supplementary Figure 3G–N,G'–L', Supplementary Table 4). These results suggest that an expanded HZ was not associated with decreased cell death. However, the percentage of apoptotic cells in the HZ in PB-treated group was less than that of the control group (Figures 3G–J, Supplementary Table 4), suggesting that PB might delay the ossification of long bones.

These data indicate that the expansion of the HZ in the PB growth plate is not caused by increased proliferation or decreased apoptosis of chondrocytes in RZ or PZ. Therefore, the shortened length of long bone may be due to the decreased proliferation, and the hypertrophic zone phenotype may be caused by the ossification defect.

### PB exposure delays ossification of long bones and mineralization in MC3T3-E1 cultures

**Figure 4:**
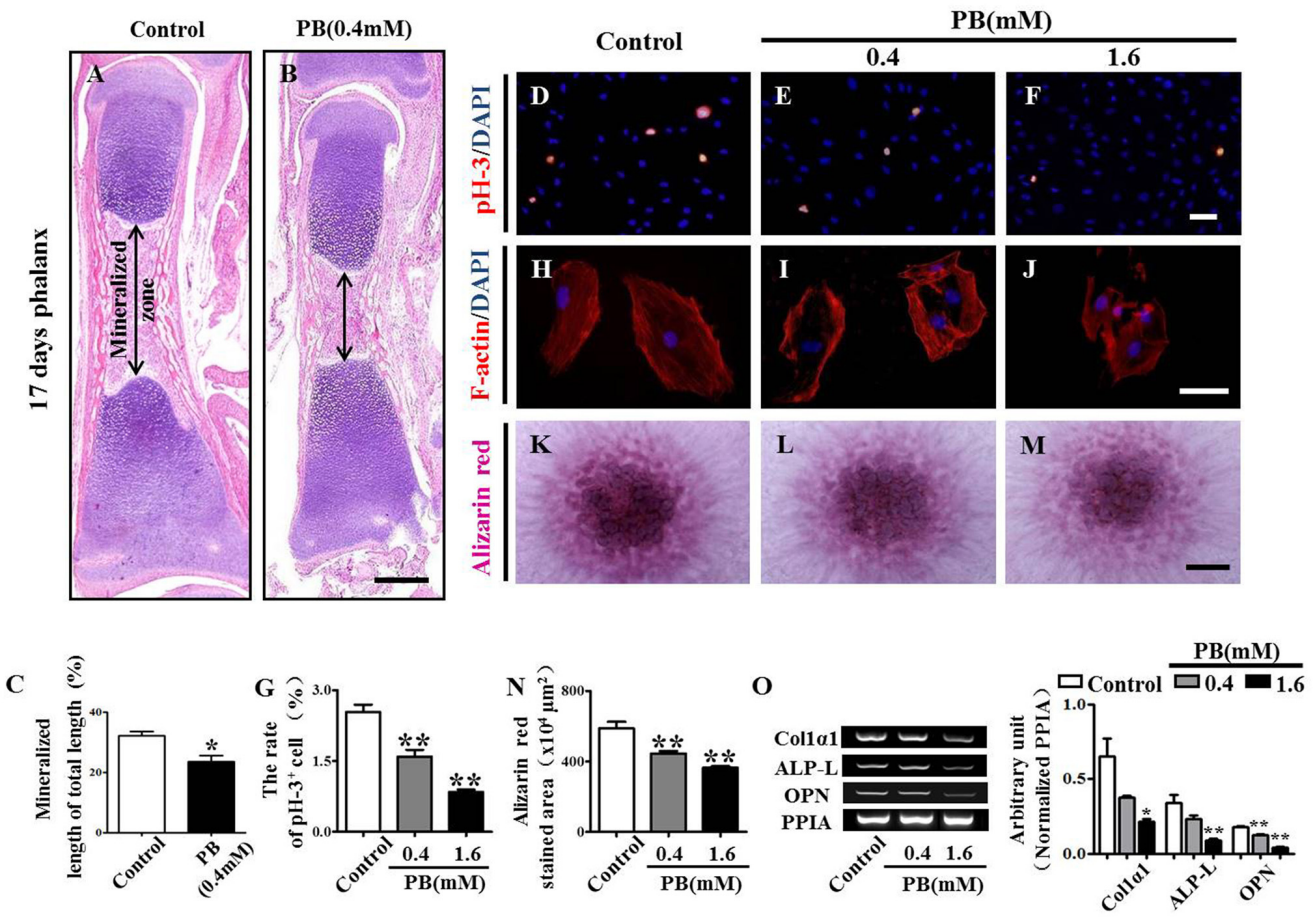
**H&E staining of phalanges and the assessment of PB treatment effects on the proliferation and mineralization of MC3T3-E1 cells**. Longitudinal sections were produced from the phalanges of day 17 chick embryos exposed to PB. MC3T3-E1 cells were used to determine the effect of PB on osteoblastic cell proliferation. **(A,B)** Representative images of H&E stained phalanx sections from control **(A)** and PB-treated MC3T3-E1 cells **(B)** at day 17. **(C)** Bar graphs comparing the rate of the mineralized length to the total length of phalanges of control and PB treatment groups. **(D–F)** pH3^+^ immunofluorescence staining was performed on MC3T3-E1 cells to show cell proliferation in the absence or presence of PB. **(G)** Bar graph comparing the number of pH3^+^ MC3T3-E1 cells in the control and PB cultures. **(H–J)** F-actin fluorescence staining was performed in the control and PB-treated MC3T3-E1 cells. **(K–M)** Alizarin red staining was performed in the control and PB-treated MC3T3-E1 cells after a 7-day culture. **(N)** Bar graph comparing the alizarin red^+^ area in the control and PB-treated MC3T3-E1 cell cultures. **(O)** Semi-quantitative RT-PCR and bar graph showing the expression of Col1α1, ALP-L, and OPN in MC3T3-E1 cells following PB treatment. Scale bars = 400 μm in **(A,B)**, 25 μm in **(D–F)**, **(H–J)**, and 500 μm in **(K–M)**. *P < 0.05, **P < 0.01.

To explore whether the expanded hypertrophic zone resulted from a defect in ossification, we performed H&E staining on the vertical sections of phalanges and measured the area of the mineralized zone of the phalanges (Figures 4A–C, Supplementary Table 4). We observed that the mineralized zone of the phalanges was narrowed by the PB treatment, suggesting that PB natively affects the ossification of long bones.

To further determine the inhibitory effect of PB on ossification, we used MC3T3-E1 cells to investigate the possible PB effects on the function of osteoblasts. We already showed that PB inhibited MC3T3-E1 cell viability in a dose-dependent manner. This inhibitory effect of PB was further confirmed by the pH3 immunofluorescent staining of PB-treated MC3T3-E1 cells (Figures 4D–G, Supplementary Table 5). Actin polymerization was determined using phalloidin staining. We observed that PB remarkably weakened the actin polymerization and caused the cells to lose their polarities (Figures 4H–J). The high-density micromass culture system stained with alizarin red dye also showed that PB significantly decelerated the mineralization of MC3T3-E1 cultures compared with that of the control culture (Figures 4K–N, Supplementary Table 5). Furthermore, we examined the expression of osteoblast markers using semi-quantitative RT-PCR analysis, including Col1α1, ALP-L, and OPN (Figure 4O, Supplementary Table 5). Taken together, these data reveal that the PB treatment caused a delay in the ossification of long bones and inhibited the cytoskeletal organization of MC3T3-E1 cells.

### PB treatment inhibits angiogenesis *in vivo* and *in vitro*

**Figure 5:**
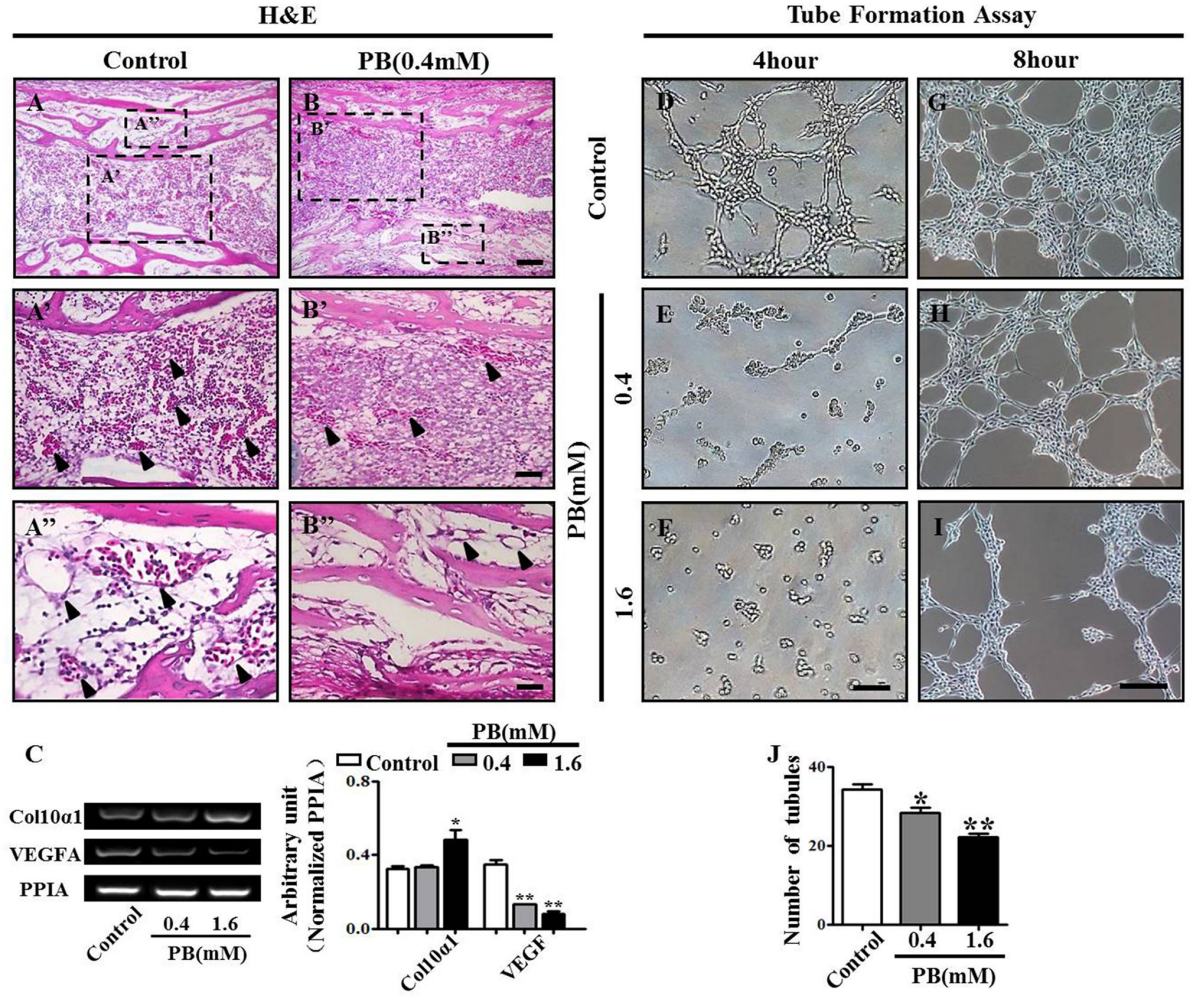
**PB treatment effects on the vascularization of long bones and the tube formation of HUVECs**. Longitudinal sections were produced from the phalanges of day 17 chick embryos exposed to PB. HUVECs were used to determine the effect of PB on tube formation. **(A,B)** Phalanges of control **(A)** and PB-treated HUVECs **(B)** stained with H&E to indicate vascularization. **(A',B')** Representative high-magnification images from the sites indicated by dotted squares in **(A,B)**. Arrows in **(A'**,**B')** indicate the vascularization in marrow cavity **(A')** and bone collar **(B')**. **(A”,B”)** Representative high-magnification images from the sites indicated by dotted squares in **(A,B)**. Arrows in **(A”,B”)** indicate the vascularization in the marrow cavity **(A”)** and bone collar **(B”)**. **(C)** Semi-quantitative RT-PCR and bar chart showing the expression of Col10α1 and VEGFA in the growth plate following PB treatment. **(D–I)** Light microscopy images of tube formation in HUVECs following 0.9% sterile saline or PB treatment for 4 h **(D–F)** or 8 h **(G–I)**. **(J)** Bar graph showing the average tube numbers in the control and PB cultures. Scale bars = 50 μm in **(A,B)**, 25 μm in **(A',B')**, **(A”,B”)**, and 200 μm in **(D–I)**. *P < 0.05, **P < 0.01.

To discover whether the delayed ossification phenotype results from a defect of vascular invasion, we performed H&E staining on the marrow cavity of phalanges at day 17 and found that the number of blood vessels (arrowheads in Figures 5A'–B”) in the PB-treated group was less than that in the control group. This observation showed that PB delayed vascular invasion in long bones. Then, we isolated the growth plates from the 14-day-incubated embryos and cultured those in the absence or presence of PB for 72 h. The expression of Col10α1 and VEGFA was determined using semi-quantitative RT-PCR. The former is a specific marker of hypertrophic chondrocytes, and the latter is an angiogenesis-related gene (Figure 5C, Supplementary Table 5).

**Figure 6:**
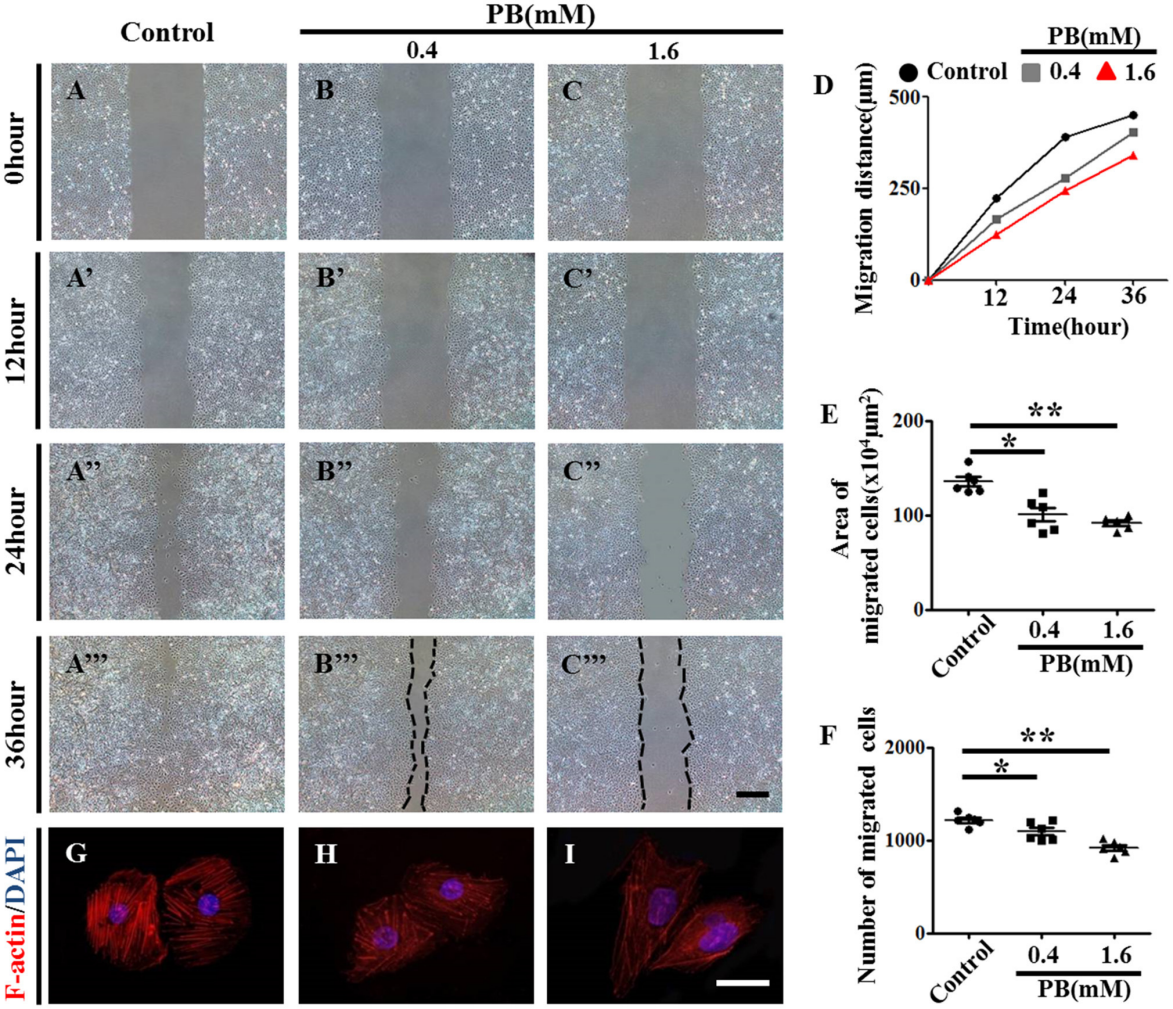
**Scratch-wound assay to investigate the effects of PB treatment on the migration of HUVECs**. **(A–C)** Representative images of the scratch-wound assay in HUVECs at 0-h incubation from the control **(A)**, 0.4 mM PB-treated **(B)**, and 1.6 mM PB-treated groups **(C)**. **(A'–C”')** Representative images of the scratch-wound assay in HUVECs at 12 h **(A'–C')**, 24 h **(A”–C”)**, and 36 h incubation **(A”'–C”')** from the control, 0.4 mM PB-treated, and 1.6 Mm PB-treated groups. **(D)** Bar graph showing the migration distances of HUVECs along with incubation time in the presence or absence of PB. **(E)** Bar graph showing the area of HUVEC migration at 36 h in the presence or absence of PB. **(F)** Bar graph showing the number of migrated HUVECs at 36 h in the presence or absence of PB. **(G–I)** F-actin fluorescence staining was performed on HUVECs treated with 0.9% sterile saline or PB for 24 h. Scale bars = 500 μm in **(A–C)**, **(A'–C')**, **(A”–C”)**, **(A”'–C”')**, and 25 μm in **(G–I)**. *P < 0.05, **P < 0.01.

To further explore the role of PB in angiogenesis, we used HUVECs to conduct tube formation assays and scratch-wound assays. First, the tube formation assay showed that PB treatment restricted tube formation compared to that of the control group (Figures 5D–J, Supplementary Table 5). Meanwhile, the scratch-wound assay showed that PB treatment for 12 h (Figures 6A'–C'), 24 h (Figures 6A”–C”) or for 36 h (Figures 6A”'–C”') inhibited the cell migration distance toward the midline along with incubation time in comparison with that of the control group (Fig. 6D). Both the area (Figure 6E, Supplementary Table 5) and number of migrated cells toward the midline (Figure 6F, Supplementary Table 5) were reduced by 36 h of PB treatment. This inhibitory effect of PB was further confirmed by F-actin immunofluorescence staining of PB-treated HUVECs, and cytoskeletal organization was markedly weakened by PB treatment (Figs. 6G-I). Together, these findings suggest that the PB treatment indeed impaired vascular invasion and inhibited the migration ability of HUVECs.

### PB treatment affects angiogenesis in chick YSM and CAM

**Figure 7:**
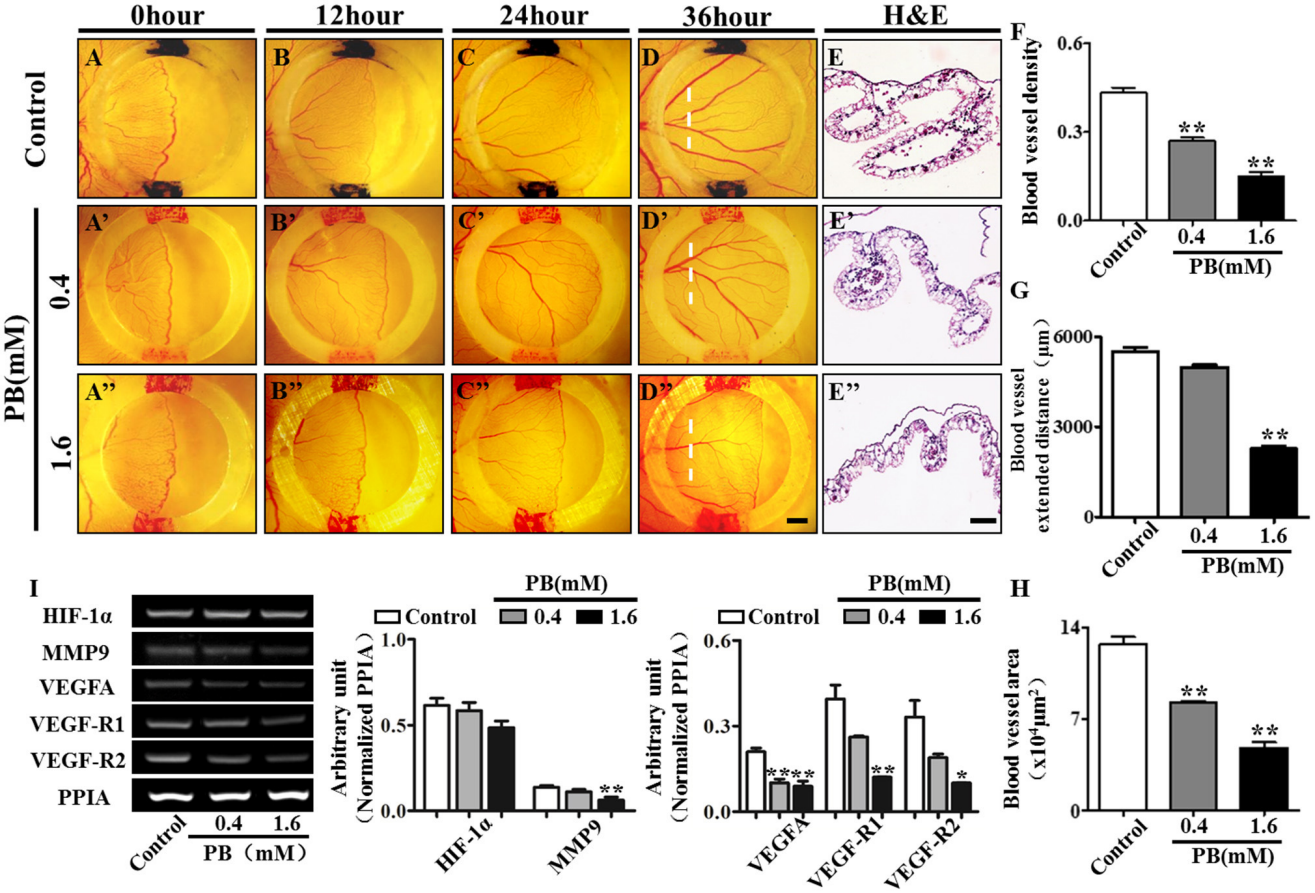
**PB treatment effects on angiogenesis in YSM**. The colored ink marks on the silicon rings define the leading edges of the developing blood vessel plexus in the YSM of 2.5-day chick embryos. **(A–D)** Representative images of the appearance of the blood vessel plexus after 0 h **(A)**, 12 h **(B)**, 24 h **(C)**, and 36-h incubation **(D)** in the control group. **(A'–D')** Representative images of the appearance of the blood vessel plexus after 0 h **(A')**, 12 h **(B')**, 24 h **(C')**, and 36-h incubation **(D')** in the 0.4 mM PB-treated group. **(A”–D”)** Representative images of the appearance of the blood vessel plexus after 0 h **(A”)**, 12 h **(B”)**, 24 h **(C”)**, and 36-h incubation **(D”)** in the 1.6 mM PB-treated group. **(E–E”)** Representative H&E stained transverse sections of the YSM from the sites indicated by dotted lines in **(D,D”)**. **(F)** Bar graph comparing the blood vessel density of the YSM at 36 h between the control and PB-treated groups. **(G)** Bar graph comparing the blood vessel extent in the YSM at 36 h between the control and PB-treated groups. **(H)** Bar graph comparing the blood vessel area of the YSM at 36 h between the control and PB-treated groups. **(I)** Semi-quantitative RT-PCR and bar chart showing the expression of HIF-1α, MMP9, VEGFA, VEGF-R1, and VEGF-R2 in the YSM following PB treatment. Scale bars = 1 mm in **(A–D)**, **(A'–D')**, **(A”–D”)**, and 200 μm in **(E–E')**. *P < 0.05, **P < 0.01.

To investigate the effect of PB on angiogenesis *in vivo*, we used the YSM angiogenesis model. PB or 0.9% sterile saline (control) was administered to the silicon rings. These rings are useful in retaining PB and sterile saline in one place on the YSM. The starting point of the YSM blood vessels was marked with red/black inks on the silicon rings and was kept constant in all replicates. We found that PB treatment significantly decreased the expansion velocity of the blood vessel plexus compared with that of the control embryos (Figures 7A–D,A'–D',A”–D”). This was indicated by the leading edges of the control blood vessel plexus reaching the rings after incubation for 24 h and reaching beyond the rings after incubation for 36 h. The blood vessel density was significantly decreased in PB-treated vessels after incubation for 36 h (Figure 7F, Supplementary Table 6). The extended distance of blood vessels was inhibited when exposed to PB for 36 h (Figure 7G, Supplementary Table 6). The area of transverse sections occupied by blood vessels was significantly decreased in the PB treated group compared to that of the control group (Figures 7E–E”,H, Supplementary Table 6). Furthermore, RT-PCR data showed that PB treatment down-regulated angiogenesis-related genes, including HIF-1α, MMP9, VEGFA, VEGF-R1, and VEGF-R2 (Figure 7I, Supplementary Table 6).

To further verify the observation above in a chick YSM model, we also used CAM, another angiogenesis model. Again we observed that the blood vessel density in chick CAM was suppressed after treatment with PB for 48 h (Supplementary Figures 4A–D,A'–C', Supplementary Table 6). Furthermore, RT-PCR data showed that PB treatment down-regulated the angiogenesis-related genes HIF-1α, VEGFA and VEGF-R1 (Supplementary Figure 4E, Supplementary Table 6). These data suggest that PB treatment indeed suppressed angiogenesis in the chick YSM and CAM models.

## Discussion

AEDs are extensively used for pregnant women with epilepsy to control their seizures and avoid complications. If withdrawn, there is a high risk of mortality for both the mother and the fetus, and intranasal hemorrhage and heart rate alterations often occur in the fetus (Ahir and Pratten, 2014). Because the chronic administration of AEDs can lead to a variety of disorders of bone and mineral metabolism, a wider range of AED application has been limited. It is worth noting that the impact of AED treatment on endochondral ossification during fetal osteogenesis is still unclear, although people have noted the adverse effect of long-term use of AEDs on bone development (Hahn et al., 1978). Therefore, it is necessary to reveal the mechanism behind these effects of AEDs on osteogenesis during embryogenesis to avoid the harmful side effects of AED application in a clinical setting. Here, we focused on the effect of PB, a commonly prescribed AED in the clinic, on bone development during embryogenesis.

Endochondral ossification involves two critical steps: the initial formation of a cartilage model and the eventual replacement of the model with vasculature, osteoblasts, osteoclasts and bone matrix. That both processes work in proper coordination is essential for normal bone development. In this study, we observed that PB treatment caused shorter long bones in chick embryos, including the phalanx, tibia, radius and ulna (Figure 1, Supplementary Figure 2). We assumed that there might be two possibilities to give rise to the shortened length of chick long bones. The formation of a proper cartilage model is a prerequisite for normal endochondral ossification (Knudson and Knudson, 2001). Therefore, the first possibility that may cause shortened long bones is the small cartilage template induced by PB treatment. This hypothesis is supported by the experiments using the high-density micromass culture system of limb bud mesenchymal cells, where we demonstrated that PB treatment impaired the capacity of mesenchymal cells to differentiate into chondrocytes, and the expression of chondrogenesis-related genes SOX9 and Col2α1 was decreased following PB treatment (Figure 2). SOX9 plays an essential role in early chondrogenesis, and Col2α1 is expressed specifically in chondrocytes (Kosher et al., 1986; Akiyama et al., 2002). In addition, the cell proliferation of chondrocytes in the growth plate decreased in the RZ and PZ following PB treatment (Figures 3D–F,D'–E' and Supplementary Figures 3A–F,A'–D'). These observations are in accordance with human studies in which AEDs caused a deficiency of vitamin D, which is involved in cell proliferation and differentiation (Wilson et al., 2003; Rovner and O'Brien, 2008). These findings undoubtedly confirm our assumption. The other possible explanation for the shorter long bones is that the process of mineralization was defective. We found that mineralization happened later than normal following PB treatment. This conjecture is based on our observation that PB treatment led to decreased apoptosis of HZ in the growth plate (Figures 3G–J). Apoptosis in the HZ is a necessary process for mineralization (Ornitz and Marie, 2002). We also found that PB treatment caused an extended HZ (Figures 3A–C). This phenotype might not be induced by increased proliferation or by decreased apoptosis of chondrocytes in the RZ and PZ (Figures 3D–J,D'–E' and Supplementary Figures 3A–N,A'–L'). Hence, we speculate that the decreased apoptosis and extended length of HZ in PB-treated bone may be caused by an ossification defect. AED treatment causes the blockage of calcium channels, which eventually leads to a loss in bone mineral density (Hernández-Díaz and Levin, 2014), might explain the phenotype in this study.

Bone ossification requires a delicate balance between bone formation of osteoblasts and bone resorption of osteoclasts (Huang et al., 2014). Being devoid of walking and bearing weight, the number of osteoclasts during embryogenesis is considerably less than that after birth (Cheng et al., 2016). Therefore, we used MC3T3-E1 cells to further determine whether PB treatment inhibited ossification of long bones. We observed that the proliferation of MC3T3-E1 cells was inhibited by PB (Figures 4D–G). In addition, PB treatment disrupted the cytoskeleton of MC3T3-E1 cells and caused the cells to lose their polarity (Figures 4H–J), which might cause an inhibitive effect on cell vitality and indirectly lowered the proportion of osteoblasts that differentiated into osteocytes [8]. We further confirmed this result by alizarin red staining, in which we observed that PB treatment decelerated the deposition of calcium salt and ossification. Furthermore, PB treatment reduced the expression of Col1α1, ALP-L, and OPN in MC3T3-E1 cells, and the reduced expression of osteoblast markers also indicated the inhibition of ossification (McKee et al., 1992; Miao and Scutt, 2002; Ornitz and Marie, 2002) (Figures 4K–O).

It is reported that PB treatment can impair cardiovascular development, which naturally reminds us to detect the effects of PB on blood vessels since angiogenesis plays a crucial role during bone ossification and formation (Bath and Scharfman, 2013; Cheng et al., 2016). In this study, we found that PB treatment suppressed vascular invasion in the development of long bones. As an important regulator of blood vessel invasion into the cartilage model, VEGFA expression in HZ was down-regulated by PB treatment (Figures 5A–C; Pfander et al., 2004; Zelzer and Olsen, 2005), Therefore, we speculate that PB treatment might affect the angiogenesis during bone development, which in turn reduces bone mineralization. To verify this assumption, we firstly performed tube formation assays and scratch-wound assays using HUVECs. The results indicated that PB treatment suppressed tube formation (Figures 5D–J) and restrained the migration of HUVECs. These results were further confirmed by the observation that PB treatment disrupted the HUVECs' cytoskeleton, which plays an important role in cell migration (Figure 6). Next, to identify the effect of PB on angiogenesis *in vivo*, we used chick embryonic YSM and CAM models to further investigate whether PB affected angiogenesis. The results showed that PB treatment restricted angiogenesis and decreased the expression of angiogenesis-related genes, HIF-1, MMP9, VEGFA, VEGF-R1, and VEGF-R2, which indeed could support the above observations (Figure 7, Supplementary Figure 4). Those angiogenesis-related genes play crucial roles in angiogenesis during bone growth and development (Araldi and Schipani, 2010; Olivares-Navarrete et al., 2013). Both *in vivo* and *in vitro* experiments on angiogenesis appear to validate our above hypothesis.

It should be noted that the dosage of PB we employed in this experiments is higher than the average dosage of PB used in adults, which is limited to 60–240 mg/day to minimize the side effects of PB. It was reported that PB administered with similar protocols up to 0.1 mM caused some heart defects in embryonic chick cardiomyocyte cultures (Ahir and Pratten, 2014). Congenital heart defects were usually considered as the major side effects of PB while the digital, craniofacial and growth retardation as its minor side effects, that was to say, the teratogenetic sensitivity of bone should be lower than that of heart. With a purpose of establishing an acute teratogenic model to investigate its effects on embryonic skeletogenesis, a dosage of 0.4 mM PB was mainly employed in this experiment, which was within an acceptable range. It was also supported by the study that 0.4 mM PB affected hormonally mediated bone resorption processes of the cultured fetal rat long bone (Hahn et al., 1978). Moreover, CCK-8 assay was used to detect the effects of PB exposure on cell viability, in order to figure out the possible concentration which affected both MC3T3-E1 and HUVECs. Generally, the earlier the embryos are exposed to the drugs, the higher the mortality will be, Therefore, the chick embryos were exposed to PB at a later stage in this experiments in case causing a high mortality. HH10 was selected as the first PB exposure time point, when the embryos showed much stronger viability, while the skeletogenesis was still at the very primitive stage of neural crest. Another consideration was that PB exposure might affect cell viability in cultures, hence the inhibition of cell viability and apoptosis caused by PB should be taken into account when discussing the mechanism related to the PB related phenotypes observed *in vitro*. In this experiment, we used chick embryos as the animal model in this experiment, with the advantage of observing angiogenesis more intuitively by using chick CAM and YSM, which is hard to manipulate by other animal models. Nevertheless, it should also be acknowledged that chick embryos have the limitations on lower uptake of chemicals from the CAM and without exrecetory pathways.

**Figure 8:**
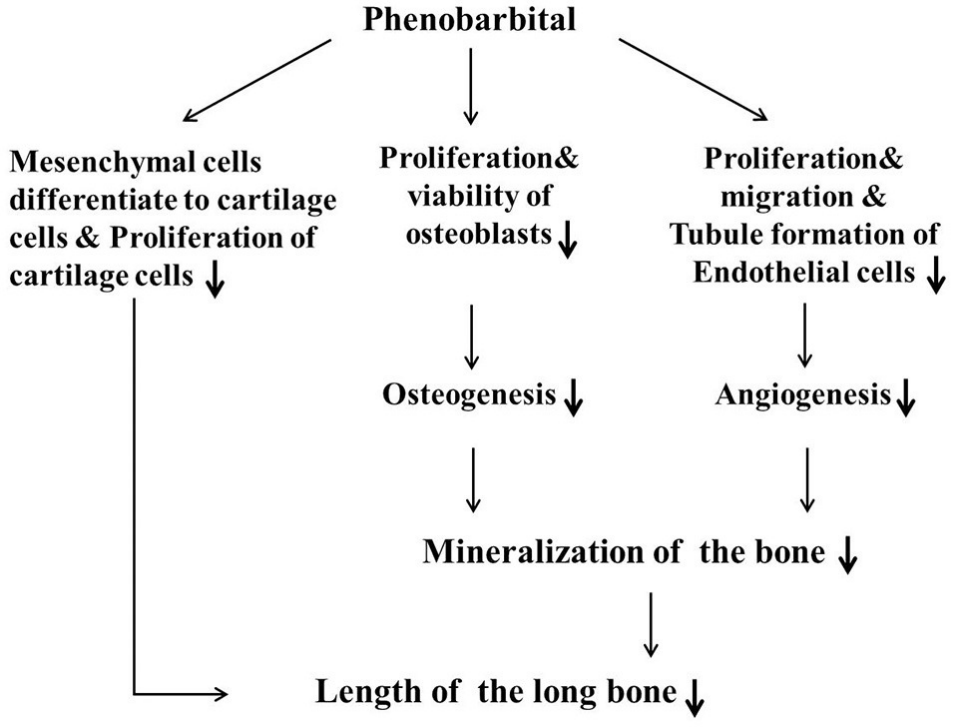
**Proposed mechanism by which PB treatment decelerates mineralization and shortens long bones during embryonic skeletogenesis**.

In summary (Figure 8), we have used a combination of *in vivo* and *in vitro* experimental approaches to demonstrate that PB treatment shortened embryonic long bones. PB treatment inhibited chondrogenesis and proliferation of chondrocytes, and later it may be influenced ossification by inhibiting the proliferation of osteoblasts and vascular invasion. Further experimentation is required to explore the molecular mechanisms underlying PB's effects on bone development.

## Author contributions

YY conducted experiments, researched data, wrote the majority of the manuscript. RY, HL, and JC conducted part of experiments. ZM carried out the histological lab work. GW and MC contributed to discussion. XC contributed to discussion and reviewed the manuscript. XY researched data, contributed to discussion, and wrote the majority of the manuscript. All authors gave final approval for publication.

### Conflict of interest statement

The authors declare that the research was conducted in the absence of any commercial or financial relationships that could be construed as a potential conflict of interest.

## Supplementary material

Supplementary Figure 1 **Sets of primers used for RT-PCR in this study**.

Supplementary Figure 2 **PB treatment effects on osteogenesis of long bones**. Alcian blue and alizarin red staining were performed in day-17 chick embryos treated with PB for 15.5 days. **(A–A”')** Whole embryo **(A)**; representative images of the appearance of day-17 chick embryos' vertebra **(A')**, radius and ulna **(A”)**, and tibia **(A”')** from the 0.9% sterile saline treatment group (control). **(B–B”')** Whole embryo treated with 0.4 mM PB **(B)**. Representative images of the appearance of day-17 chick embryos' vertebra **(B')**, radius and ulna **(B”)**, and tibia **(B”')** from the PB treatment groups. **(C–E)** Bar charts comparing the rate of alizarin red^+^ length to the total length of the ulna **(C)**, radius **(D)** and tibia **(E)** between the control and PB treatment groups. **(F–H)** Bar charts comparing the length of the ulna **(F)**, radius **(G)**, tibia **(H)** between the control and PB-treated embryos. Scale bars = 5 cm in **(A,B)** and 1 cm in **(A',B')**, **(A”,B”)**, **(A”',B”')**.

Supplementary Figure 3 **PB treatment effects on the cell cycles and the apoptosis of growth plates in phalanx**. Histological analysis of epiphyseal growth plates in the phalanges of 17-day-old chick embryos exposed to PB. **(A–D)** pH3^+^ immunofluorescence of phalanges of the control **(A,B)** and PB-treated embryos **(C,D)** in the RZ or PZ of the growth plate. **(A'–D')** Representative high-magnification images from the sites indicated by dotted squares in **(A–D)**. **(E–F)** Bar charts comparing the rate of pH3^+^ cells in the RZ **(E)** or PZ **(F)** of the growth plate between the control and PB-treated phalanges. **(G–J)** RZ or PZ of control **(G,H)** and PB-treated phalanges **(I,J)** stained with TUNEL. **(G'–J')** Representative high-magnification images from the sites indicated by dotted squares in **(G–J)**. **(K,L)** Representative images of the negative control of TUNEL staining in the RZ **(K)** or PZ **(L)** of the growth plate. **(K',L')** Representative high-magnification images from the sites indicated by dotted squares in **(K,L)**. **(M,N)** Bar charts comparing apoptotic chondrocytes of the RZ **(M)** or PZ **(N)** in the growth plate between the control and PB-treated phalanges. Scale bars = 50 μm in **(A,B)**, 25 μm in **(A',B')**, 50 μm in **(G–L)**, and 25 μm in **(G'–L')**.

Supplementary Figure 4 **PB treatment effects on angiogenesis in the CAM. (A–C)** Representative images of the vessel plexuses in the CAM, which were treated with 0.9 sterile saline (control, **A)**, 0.4 mM PB **(B)**, or 1.6 mM PB **(C)** for 48 h. **(A'–C')** Representative high-magnification images from the sites indicated by dotted squares in **(A–C)**. **(D)** Bar graph showing the comparison of blood vessel densities in the CAM model following treatment with different concentrations of PB. **(E)** Semi-quantitative RT-PCR and bar graph showing the expression of HIF-1α, VEGFA, and VEGF-R1 in the YSM model following PB treatment. Scale bars = 1 cm in **(A–C)** and 3 mm in **(A'–C')**.

Supplementary Table 1 **The data of Figures 1I–J, Supplementary Figures 2C–H**. The results are presented as the mean ± SD. All comparisons between groups were made using ANOVA or Student's *t*-test. **P* < 0.01, ***P* < 0.05.

Supplementary Table 2 **The data of Figures 1K–L**. The results are presented as the mean ± SD. All comparisons between groups were made using ANOVA or Student's *t*-test. **P* < 0.01, ***P* < 0.05.

Supplementary Table 3 **The data of Figures 2G,H**. The results are presented as the mean ± SD. All comparisons between groups were made using ANOVA or Student's *t*-test. **P* < 0.01, ***P* < 0.05.

Supplementary Table 4 **The data of Figures 3C,F,J, Figures 4C and Supplementary Figures 3E,F,M,N**. The results are presented as the mean ± SD. All comparisons between groups were made using ANOVA or Student's *t*-test. **P* < 0.01, ***P* < 0.05.

Supplementary Table 5 **The data of Figures 4G,N,O, Figures 5C,J and Figures 6D–F**. The results are presented as the mean ± SD. All comparisons between groups were made using ANOVA or Student's *t*-test. **P* < 0.01, ***P* < 0.05.

Supplementary Table 6 **The data of Figures 7F–I, Supplementary Figures 4D,E**. The results are presented as the mean ± SD. All comparisons between groups were made using ANOVA or Student's *t*–test. **P* < 0.01, ***P* < 0.05.
