# Supplementary figures and images for: Exposure to Excess Phenobarbital Negatively Influences the Osteogenesis of Chick Embryos
Source: Front Pharmacol. 2016 Sep 30;7:349. doi: 10.3389/fphar.2016.00349 (PMC5044464; doi:10.3389/fphar.2016.00349)

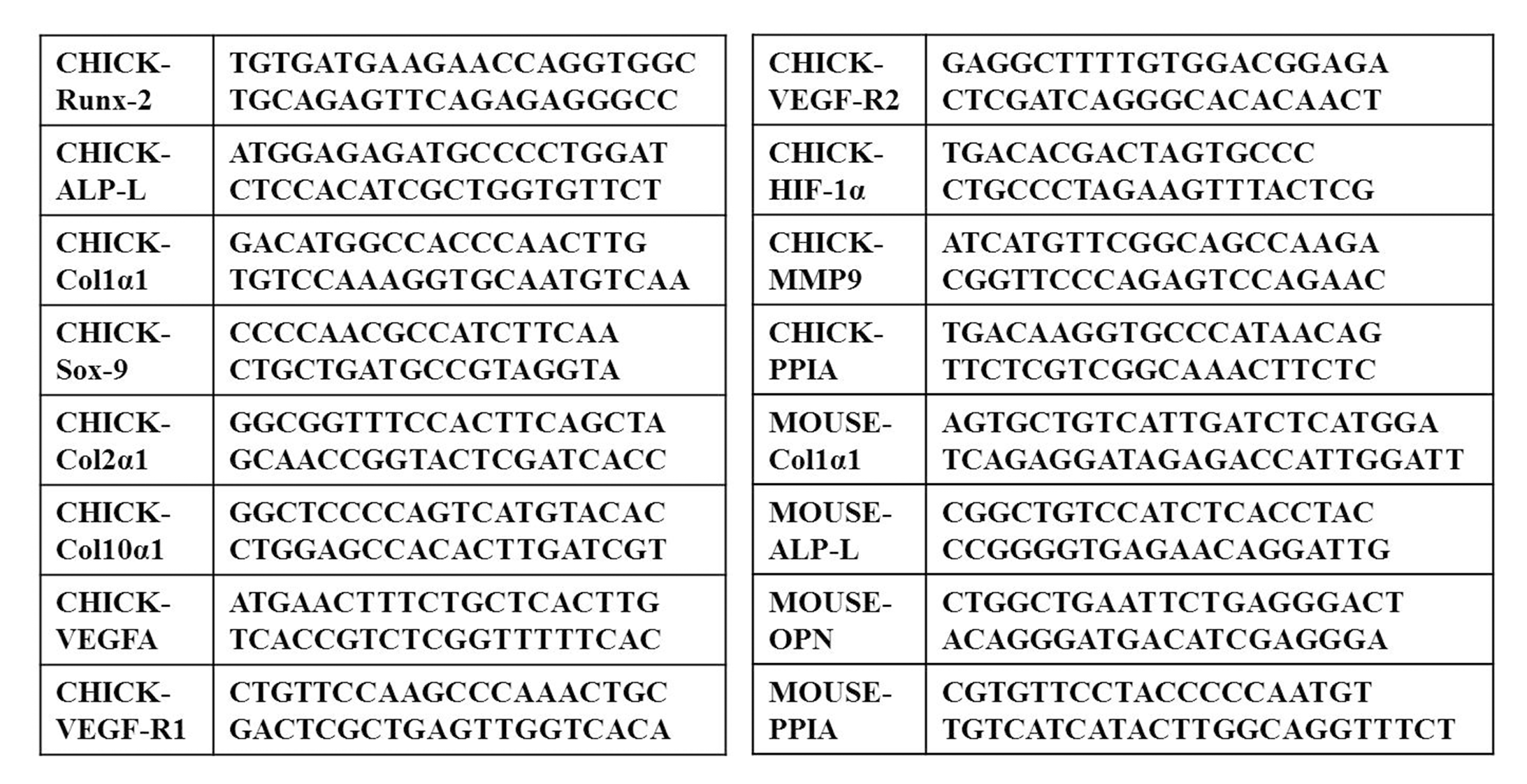

Supplement: Supplementary Figure 1 — Sets of primers used for RT-PCR in this study. [file Image1.TIF]

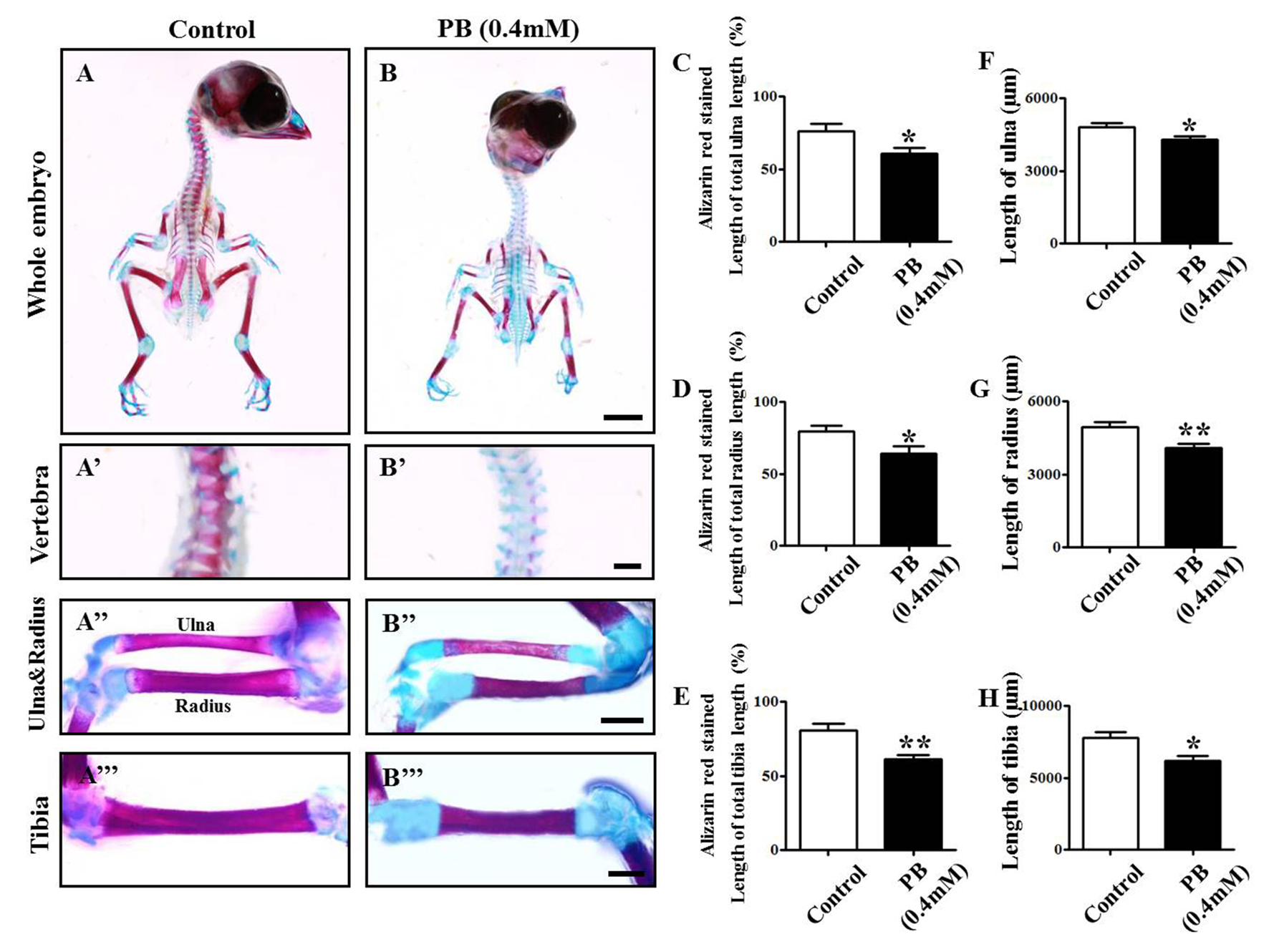

Supplement: Supplementary Figure 2 — PB treatment effects on osteogenesis of long bones. Alcian blue and alizarin red staining were performed in day-17 chick embryos treated with PB for 15.5 days. (A–A”') Whole embryo (A); representative images of the appearance of day-17 chick embryos' vertebra (A'), radius and ulna (A”), and tibia (A”') from the 0.9% sterile saline treatment group (control). (B–B”') Whole embryo treated with 0.4 mM PB (B). Representative images of the appearance of day-17 chick embryos' vertebra (B'), radius and ulna (B”), and tibia (B”') from the PB treatment groups. (C–E) Bar charts comparing the rate of alizarin red+ length to the total length of the ulna (C), radius (D) and tibia (E) between the control and PB treatment groups. (F–H) Bar charts comparing the length of the ulna (F), radius (G), tibia (H) between the control and PB-treated embryos. Scale bars = 5 cm in (A,B) and 1 cm in (A',B'), (A”,B”), (A”',B”'). [file Image2.TIF]

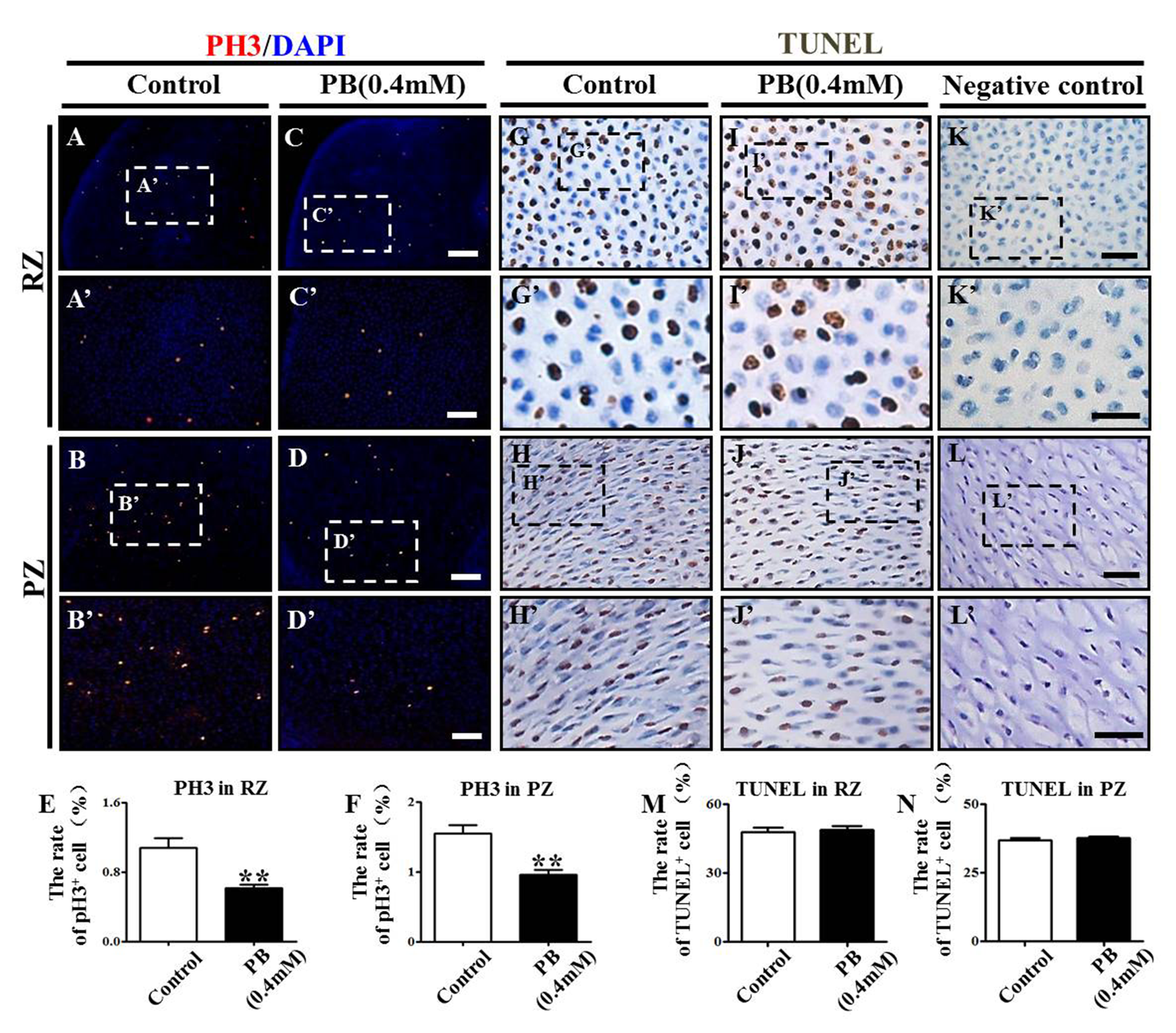

Supplement: Supplementary Figure 3 — PB treatment effects on the cell cycles and the apoptosis of growth plates in phalanx. Histological analysis of epiphyseal growth plates in the phalanges of 17-day-old chick embryos exposed to PB. (A–D) pH3+ immunofluorescence of phalanges of the control (A,B) and PB-treated embryos (C,D) in the RZ or PZ of the growth plate. (A'–D') Representative high-magnification images from the sites indicated by dotted squares in (A–D). (E–F) Bar charts comparing the rate of pH3+ cells in the RZ (E) or PZ (F) of the growth plate between the control and PB-treated phalanges. (G–J) RZ or PZ of control (G,H) and PB-treated phalanges (I,J) stained with TUNEL. (G'–J') Representative high-magnification images from the sites indicated by dotted squares in (G–J). (K,L) Representative images of the negative control of TUNEL staining in the RZ (K) or PZ (L) of the growth plate. (K',L') Representative high-magnification images from the sites indicated by dotted squares in (K,L). (M,N) Bar charts comparing apoptotic chondrocytes of the RZ (M) or PZ (N) in the growth plate between the control and PB-treated phalanges. Scale bars = 50 μm in (A,B), 25 μm in (A',B'), 50 μm in (G–L), and 25 μm in (G'–L'). [file Image3.TIF]

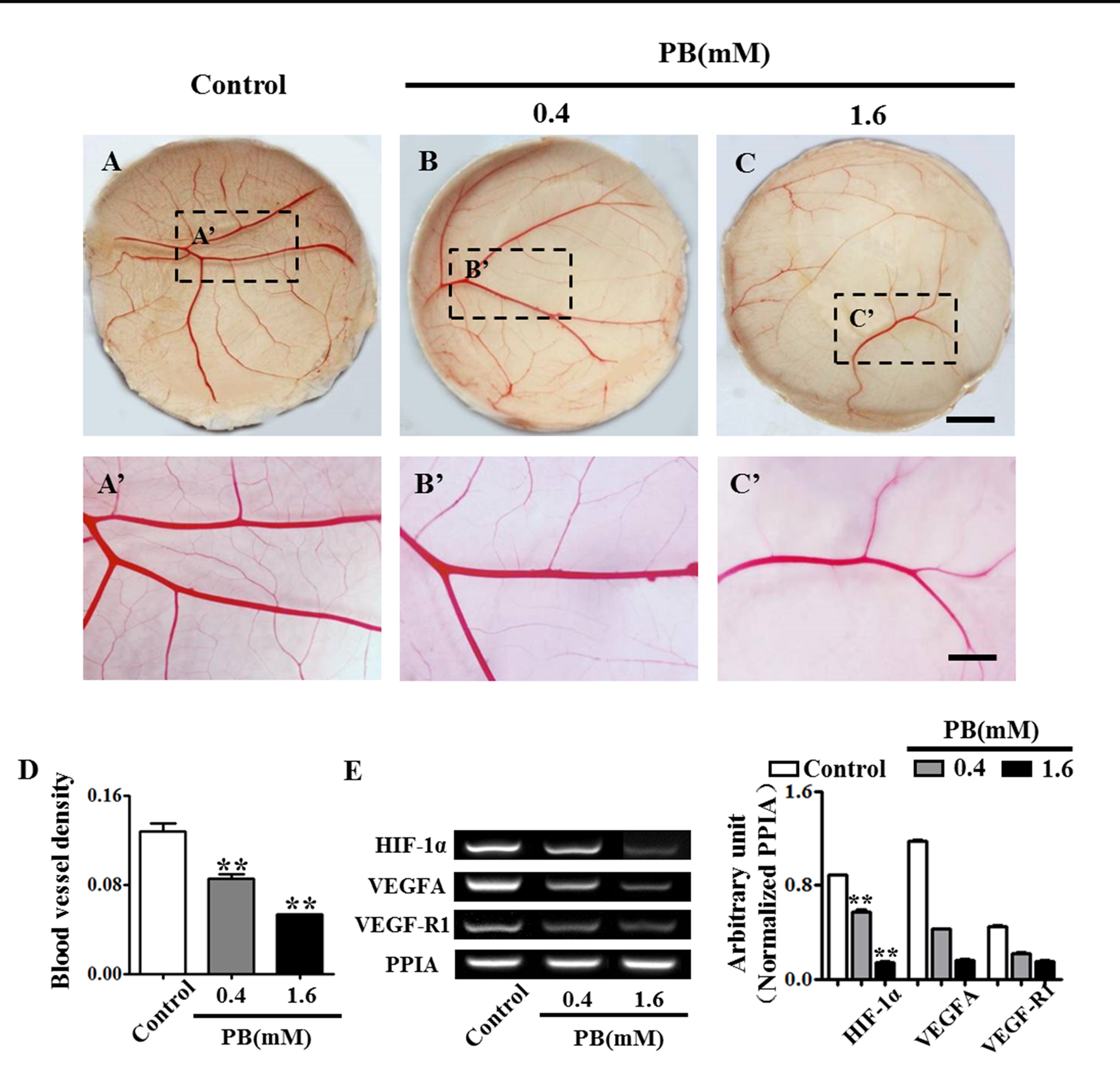

Supplement: Supplementary Figure 4 — PB treatment effects on angiogenesis in the CAM. (A–C) Representative images of the vessel plexuses in the CAM, which were treated with 0.9 sterile saline (control, A), 0.4 mM PB (B), or 1.6 mM PB (C) for 48 h. (A'–C') Representative high-magnification images from the sites indicated by dotted squares in (A–C). (D) Bar graph showing the comparison of blood vessel densities in the CAM model following treatment with different concentrations of PB. (E) Semi-quantitative RT-PCR and bar graph showing the expression of HIF-1α, VEGFA, and VEGF-R1 in the YSM model following PB treatment. Scale bars = 1 cm in (A–C) and 3 mm in (A'–C'). [file Image4.TIF]
