# Supplementary material for: Exposure to Excess Phenobarbital Negatively Influences the Osteogenesis of Chick Embryos
Source: Front Pharmacol. 2016 Sep 30;7:349. doi: 10.3389/fphar.2016.00349 (PMC5044464; doi:10.3389/fphar.2016.00349)
Supplement: Supplementary Table 1 — The data of Figures 1I–J, Supplementary Figures 2C–H. The results are presented as the mean ± SD. All comparisons between groups were made using ANOVA or Student's t-test. *P < 0.01, **P < 0.05. [file Table1.PDF]

		Control	0.4mM PB
Length of phalanges (μm)		4149.00 ± 130.10	**3658.00 ± 272.00****
Alizarin red stained length of total ulna length (%)		75.98 ± 12.39	**60.49 ± 10.59***
Alizarin red stained length of total radius length (%)		79.34 ± 9.58	**63. 97 ± 12. 96***
Alizarin red stained length of total tibia length (%)		80.56 ± 11.96	**61. 00 ± 7. 48****
Length of ulna (μm)		4810.00 ± 414.80	**4296.00 ± 359.50***
Length of radius (μm)		4959.00 ± 427.10	**4084.00 ± 454.80****
Length of tabia (μm)		7789.00 ± 983.80	**6184.00 ± 851.60****
Arbitrary unit (Normalized PPIA)	Runx-2	0.59 ± 0.01	**0.44 ± 0.83****
	ALP-L	0.83 ± 0.02	**0.24 ± 0.01****
	Collα1	0.94 ± 0.02	**0.66 ± 0.01****
