# Supplementary material for: Exposure to Excess Phenobarbital Negatively Influences the Osteogenesis of Chick Embryos
Source: Front Pharmacol. 2016 Sep 30;7:349. doi: 10.3389/fphar.2016.00349 (PMC5044464; doi:10.3389/fphar.2016.00349)
Supplement: Supplementary Table 2 — The data of Figures 1K–L. The results are presented as the mean ± SD. All comparisons between groups were made using ANOVA or Student's t-test. *P < 0.01, **P < 0.05. [file Table2.PDF]

	Control	0.1mM PB	0.2mM PB	0.4mM PB	0.8mM PB	1.6mM PB
Cell viability rate with MC3T3-E1	0.97 ± 0.04	0.94 ± 0.07	**0.88 ± 0.04***	**0.85 ± 0.06****	**0.81 ± 0.03****	**0.72 ± 0.02****
Cell viability rate with HUVECs	1.00 ± 0.05	0.98 ± 0.02	0.97 ± 0.04	**0.95 ± 0.02***	**0.92 ± 0.03****	**0.86 ± 0.01****
