# Supplementary material for: Exposure to Excess Phenobarbital Negatively Influences the Osteogenesis of Chick Embryos
Source: Front Pharmacol. 2016 Sep 30;7:349. doi: 10.3389/fphar.2016.00349 (PMC5044464; doi:10.3389/fphar.2016.00349)
Supplement: Supplementary Table 3 — The data of Figures 2G,H. The results are presented as the mean ± SD. All comparisons between groups were made using ANOVA or Student's t-test. *P < 0.01, **P < 0.05. [file Table3.PDF]

		Control	0.4mM PB	1.6mM PB
Alcian blue stained area (x10^4μm^2)		335.30 ± 26.78	**297.00 ± 9.9***	**228.20 ± 24.46****
Arbitrary unit (Normalized PPIA)	SOX-9	0.94 ± 0.04	**0.56 ± 0.01****	**0.45 ± 0.04****
	Col2α1	0.71 ± 0.13	**0.44 ± 0.08***	**0.14 ± 0.02****
