# Supplementary material for: Exposure to Excess Phenobarbital Negatively Influences the Osteogenesis of Chick Embryos
Source: Front Pharmacol. 2016 Sep 30;7:349. doi: 10.3389/fphar.2016.00349 (PMC5044464; doi:10.3389/fphar.2016.00349)
Supplement: Supplementary Table 4 — The data of Figures 3C,F,J, Figures 4C and Supplementary Figures 3E,F,M,N. The results are presented as the mean ± SD. All comparisons between groups were made using ANOVA or Student's t-test. *P < 0.01, **P < 0.05. [file Table4.PDF]

		Control	0.4mM PB
Each Length of growth plate (of total length %)	RZ	19.89 ± 1.23	18.35 ± 0.89
	PZ	51.30 ± 2.48	**43.88 ± 1.15****
	HZ	28.81 ± 2.77	**37.77 ± 0.29****
The rate of pH3+ cell in RZ (%)		1.04 ± 0.24	**0.61 ± 0.09**
The rate of PCNA+ cell in PZ (%)		16.21 ± 2.52	**9.88 ± 1.59****
The rate of pH3+ cell in RZ (%)		1.54 ± 0.29	**0.95 ± 0.16****
The rate of $TUNEL^+$ cell (%)	RZ	47.95 ± 3.56	48.82 ± 3.56
	PZ	36.94 ± 1.40	37.80 ± 1.01
	HZ	89.35 ± 3.38	**56.24 ± 4.91****
The mineralized zone of phalanges (%)		32.19 ± 2.83	**23.62 ± 3.68***
