# Supplementary material for: Exposure to Excess Phenobarbital Negatively Influences the Osteogenesis of Chick Embryos
Source: Front Pharmacol. 2016 Sep 30;7:349. doi: 10.3389/fphar.2016.00349 (PMC5044464; doi:10.3389/fphar.2016.00349)
Supplement: Supplementary Table 5 — The data of Figures 4G,N,O, Figures 5C,J and Figures 6D–F. The results are presented as the mean ± SD. All comparisons between groups were made using ANOVA or Student's t-test. *P < 0.01, **P < 0.05. [file Table5.PDF]

		Control	0.4mM PB	1.6mM PB
The rate of pH3⁺ MC3T3-E1 (%)		2.53 ± 0.39	**1.59 ± 0.35****	**0.84 ± 0.13****
Alizarin red stained area (x$10^4 \mu m^2$)		587.70 ± 65.05	**444.80 ± 24.52****	**363.90 ± 15.10****
Arbitrary unit (Normalized PPIA)	Collα1	0.65 ± 0.20	0.37 ± 0.02	**0.21 ± 0.02***
	ALP-L	0.33 ± 0.10	0.22 ± 0.04	**0.09 ± 0.01****
	OPN	0.18 ± 0.0002	**0.12 ± 0.006****	**0.04 ± 0.003****
Arbitrary unit (Normalized PPIA)	Col10α1	0.32 ± 0.02	0.33 ± 0.01	**0.48 ± 0.09***
	VEGF	0.35 ± 0.03	**0.13 ± 0.01****	**0.07 ± 0.02****
Number of tubules		34.00 ± 2.08	**28.00 ± 2.30***	**22.00 ± 1.52****
Area of migrated cells(x$10^4 \mu m^2$)		125.50 ± 9.35	**110.60 ± 9.31***	**91.67 ± 6.52****
Number of migrated cells		1215.00 ± 65	**1092.00 ± 95***	**918 ± 70****
