# Supplementary material for: Exposure to Excess Phenobarbital Negatively Influences the Osteogenesis of Chick Embryos
Source: Front Pharmacol. 2016 Sep 30;7:349. doi: 10.3389/fphar.2016.00349 (PMC5044464; doi:10.3389/fphar.2016.00349)
Supplement: Supplementary Table 6 — The data of Figures 7F–I, Supplementary Figures 4D,E. The results are presented as the mean ± SD. All comparisons between groups were made using ANOVA or Student's t–test. *P < 0.01, **P < 0.05. [file Table6.PDF]

		Control	0.4mM PB	1.6mM PB
Blood vessel density of YSM		0.43 ± 0.04	**0.27 ± 0.02****	**0.15 ± 0.02****
Blood vessel exended distance (μm)		5509.00 ± 318.30	4983.00 ± 190.20	**2274.00 ± 235.00****
Blood vessel area (x10⁴μm²)		12.68 ± 1.42	**8.22 ± 0.43****	**4.77 ± 1.13****
YSM-Arbitrary unit (Normalized PPIA)	HIF-1α	0.61 ± 0.07	0.58 ± 0.08	0.48 ± 0.07
	MMP9	0.14 ± 0.02	0.11 ± 0.02	**0.05 ± 0.001****
	VEGFA	0.21 ± 0.02	**0.10 ± 0.02****	**0.09 ± 0.03****
	VEGF-R1	0.40 ± 0.08	0.26 ± 0.01	**0.12 ± 0.004****
	VEGF-R2	0.33 ± 0.10	0.19 ± 0.02	**0.10 ± 0.004***
Blood vessel density of CAM		0.12 ± 0.01	**0.08 ± 0.01****	**0.05 ± 0.0006****
CAM-Arbitrary unit (Normalized PPIA)	HIF-1α	0.88 ± 0.01	**0.57 ± 0.02****	**0.14± 0.01 ****
	VEGFA	1.17 ± 0.02	0.43 ± 0.003	0.16± 0.01
	VEGF-R1	0.44 ± 0.01	0.22 ± 0.01	0.14± 0.01
